# MOSAIC enables selective transfer and fairness-aware generalization in cross-species DNA methylation prediction

**DOI:** 10.1093/nar/gkag822

**Published:** 2026-08-20

**Authors:** Peilin Xie, Xingchen Liu, Jiahui Guan, Zhihao Zhao, Jianzhi Wu, Tzong-Yi Lee, Junwen Wang, Lantian Yao, Bingyu Cui, Ying-Chih Chiang

**Affiliations:** School of Science and Engineering, The Chinese University of Hong Kong, Shenzhen, 2001 Longxiang Blvd, Longgang District, Shenzhen 518172, China; Kobilka Institute of Innovative Drug Discovery, School of Medicine, The Chinese University of Hong Kong, Shenzhen, 2001 Longxiang Blvd, Longgang District, Shenzhen 518172, China; Kobilka Institute of Innovative Drug Discovery, School of Medicine, The Chinese University of Hong Kong, Shenzhen, 2001 Longxiang Blvd, Longgang District, Shenzhen 518172, China; Division of Applied Oral Sciences and Community Dental Care, Faculty of Dentistry, The University of Hong Kong, Hong Kong, China; School of Science and Engineering, The Chinese University of Hong Kong, Shenzhen, 2001 Longxiang Blvd, Longgang District, Shenzhen 518172, China; Kobilka Institute of Innovative Drug Discovery, School of Medicine, The Chinese University of Hong Kong, Shenzhen, 2001 Longxiang Blvd, Longgang District, Shenzhen 518172, China; Kobilka Institute of Innovative Drug Discovery, School of Medicine, The Chinese University of Hong Kong, Shenzhen, 2001 Longxiang Blvd, Longgang District, Shenzhen 518172, China; Institute of Bioinformatics and Systems Biology, National Yang Ming Chiao Tung University, Hsinchu 300, Taiwan; Center for Intelligent Drug Systems and Smart Bio-devices (IDS2B), National Yang Ming Chiao Tung University, Hsinchu 300, Taiwan; Division of Applied Oral Sciences and Community Dental Care, Faculty of Dentistry, The University of Hong Kong, Hong Kong, China; School of Informatics, Xiamen University, Xiamen 361005, China; Faculty of Computer Science and Artificial Intelligence, Shenzhen University of Advanced Technology, Shenzhen 518107, China; School of Science and Engineering, The Chinese University of Hong Kong, Shenzhen, 2001 Longxiang Blvd, Longgang District, Shenzhen 518172, China; School of Science and Engineering, The Chinese University of Hong Kong, Shenzhen, 2001 Longxiang Blvd, Longgang District, Shenzhen 518172, China; Kobilka Institute of Innovative Drug Discovery, School of Medicine, The Chinese University of Hong Kong, Shenzhen, 2001 Longxiang Blvd, Longgang District, Shenzhen 518172, China; Institute of Bioinformatics and Systems Biology, National Yang Ming Chiao Tung University, Hsinchu 300, Taiwan

## Abstract

DNA methylation prediction increasingly relies on data collected across multiple species and modification types, yet these datasets are often highly heterogeneous, severely imbalanced, and strongly long-tailed. Separate models preserve task-specific patterns but miss transferable biological information, whereas indiscriminate data pooling can improve average performance while degrading underrepresented tasks. MOSAIC is a prompt-aware sparse mixture-of-experts framework for cross-species and cross-modification DNA methylation prediction. It combines a shared DNABERT-2 backbone with species and methylation-type prompts that guide sparse expert routing across related biological contexts, preserving task-specific specialization while enabling selective information sharing. On a 20-task benchmark spanning 4mC, 5hmC, and 6mA, MOSAIC showed the strongest overall performance-fairness profile among controlled baselines and representative predictors, achieving a higher worst-task Matthews correlation coefficient, a smaller head–tail performance gap, and a higher Jain’s fairness index. Ablation, routing, and perturbation analyses further suggested that these gains were consistent with selective sharing aligned with biologically plausible sequence and routing patterns rather than indiscriminate parameter sharing. External zero-shot evaluation further showed measurable transfer within the evaluated OOD settings. A publicly accessible web server is provided to support practical methylation prediction and routing inspection.

## Graphical Abstract

**Graphical Abstract:**
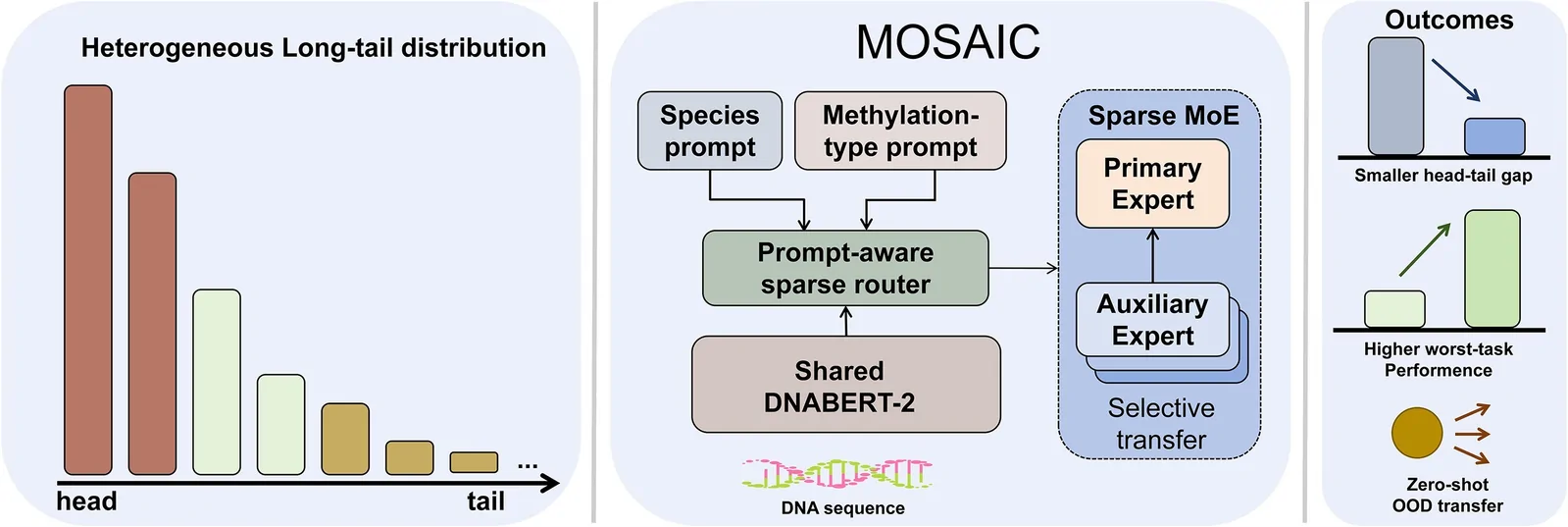

## Introduction

DNA methylation is a major epigenetic mechanism involved in genome regulation across a wide range of organisms [1]. The canonical form, 5-methylcytosine (5mC), is the most extensively studied DNA modification and has been strongly linked to gene regulation, development, and disease, particularly cancer [1, 2]. Accordingly, accurate prediction of 5mC has the potential to support regulatory analysis, biomarker discovery, and the cost-effective prioritization of candidate loci for experimental validation [2]. Other DNA modifications are also biologically important, including N4-methylcytosine (4mC), 5-hydroxymethylcytosine (5hmC), and N6-methyladenine (6mA). These marks differ in sequence context and biological function. 4mC is often associated with restriction-modification systems and bacterial epigenetic regulation [3]. 5hmC is linked to TET-mediated DNA demethylation and transcriptionally active regions, particularly in developmental and neural contexts [4, 5], and 6mA has been implicated in DNA replication, mismatch repair, and transcriptional regulation [6]. Because these modifications reflect regulatory mechanisms not captured by 5mC alone, their accurate identification remains important for advancing epigenomic research and our understanding of genome regulation.

Machine learning provides an effective computational framework for DNA methylation prediction [7, 8]. By learning reusable sequence patterns from related datasets, such models can support large-scale screening, candidate prioritization, and predictive analysis in settings where experimental coverage remains limited [9, 10]. Existing computational studies have shown that methylation-associated sequence signals can be learned effectively from DNA sequences. Earlier methods primarily relied on hand-crafted sequence features combined with conventional machine learning models, whereas more recent approaches have adopted convolutional neural networks, recurrent architectures, and transformer-based encoders to capture higher-order sequence patterns [8, 11–13]. Despite this progress, current predictors remain limited in their ability to generalize across heterogeneous datasets, particularly when species, modification types, and dataset sizes vary substantially. Many methods have been developed for individual datasets, typically representing a single modification type in a single species, and their predictive capacity is therefore constrained by dataset size. More recent general predictors attempt to share information across datasets, but such sharing is often performed only implicitly or at a coarse level [14]. As a result, potentially relevant sequence commonalities may not be transferred in a controlled manner, while differences in species background, modification type, and dataset scale are not explicitly accounted for during learning [15, 16]. These limitations reduce the effectiveness of cross-dataset learning and leave substantial room for improvement.

These limitations become especially pronounced under the severe imbalance of methylation data across species and modification types [17]. Experimental methylation profiling is often assay-specific, costly, and difficult to scale across species, tissues, and experimental conditions [7, 18], particularly when the target modification is rare, lineage-restricted, or technically difficult to resolve [18, 19]. As a result, high-quality methylation data are distributed unevenly: a small number of well-studied systems contribute most of the available samples, whereas many species and modification types remain sparsely profiled. Consequently, public methylation benchmark datasets often exhibit a long-tailed distribution, with a few high-resource subsets accounting for most samples and many others remaining low-resource [20].

Moreover, 4mC, 5hmC, and 6mA differ in taxonomic prevalence and biological role, while the corresponding benchmark datasets also vary substantially in sample size and experimental protocol. From a modeling perspective, prediction across species and modification types is therefore better viewed as a collection of related but distinct tasks [21]. Under such heterogeneous and long-tailed conditions, aggregate performance alone is not sufficient for evaluating model quality [22, 23]. Because high-resource tasks contribute disproportionately to both optimization and summary metrics, a model may achieve strong overall performance while still performing poorly on underrepresented tasks [24]. Accordingly, the relevant question in cross-task methylation prediction is not only how high performance is on average, but also how reliably that performance is maintained across tasks with markedly different biological and statistical characteristics. In this setting, fairness is naturally interpreted as task-level performance equity across the benchmark, rather than as an auxiliary or external objective [25]. It is therefore a necessary criterion for determining whether cross-task transfer is genuinely effective across the heterogeneous task landscape as a whole.

Therefore, the central challenge in building a high-performing DNA methylation prediction model is not simply whether information should be shared across tasks, but how such sharing can be achieved without overwhelming minority tasks or erasing task-specific structure [26]. This creates a fundamental modeling tension: treating tasks independently preserves task-specific structure but forfeits potentially useful transfer, whereas naive multi-task learning enables transfer but may induce negative transfer when phylogenetic relatedness, biological context, and dataset size differ substantially [27, 28]. What is needed, therefore, is a framework that preserves the benefits of shared sequence modeling while enabling selective, structured, and biologically informed transfer across tasks [29].

To address this challenge, we propose **MOSAIC** (Mixture of Sparse Adaptive experts for Interpretable Cross-context transfer), a prompt-aware sparse mixture-of-experts framework designed for selective transfer in cross-species and cross-modification methylation prediction. Rather than relying on uniformly shared learning, MOSAIC is designed to regulate when and how cross-task knowledge should be transferred, so that reusable sequence information can be retained without overwhelming minority tasks or erasing task-specific structure. Concretely, a shared DNABERT-2 backbone provides a common sequence representation space, while a species prompt and a methylation-type prompt jointly encode biological context and guide sparse expert routing [30, 31]. Task specialization is then preserved separately through task-aligned gating supervision over the expert pool. This design preserves task specialization while enabling targeted knowledge sharing across related biological contexts [32]. Accordingly, MOSAIC is designed not only to improve predictive performance under substantial biological heterogeneity, but also to ensure that these gains remain distributed more consistently across the heterogeneous task landscape. We therefore evaluate the framework not only with conventional predictive metrics, but also from the perspective of task-level fairness. The results show that MOSAIC improves both overall predictive performance and cross-task fairness. Routing and perturbation analyses further suggest that the learned transfer behavior is structured and biologically interpretable.

## Materials and methods

### Dataset

In this study, we used the same benchmark as UniMethylNet [8]. It comprises 20 publicly available component datasets covering three DNA methylation types: 4mC, 5hmC, and 6mA. These datasets were assembled from the resources reported by Lv *et al*. and Zeng and Liao [33, 34]. The data were obtained from GEO [35], MethSMRT [36], MDR [37], and NCBI [38], and span a broad taxonomic range, including plants, animals, fungi, bacteria, and protists.

Each sample was represented by a fixed 41-bp sequence window centered on the candidate methylation site. Positive samples corresponded to reported methylated sites, whereas unreported samples corresponded to sites unreported for methylation with the same candidate central base, rather than to sites with experimentally validated absence of methylation. The source-specific descriptions of unreported samples and the unified terminology adopted here are summarized in Supplementary Table S1. Following the original benchmark design, each component dataset was balanced at a 1:1 positive-to-unreported ratio, and all sequences were represented in the same fixed-length format [8]. To examine this limitation without altering the original benchmark, additional simulated imbalance test sets were constructed at 1:5 and 1:10 positive-to-unreported ratios; their construction and validation are reported in Supplementary Table S1. To ensure direct comparability with previous studies, we used the same preprocessing and partitioning protocol as UniMethylNet. Redundant sequences within each component dataset were removed using CD-HIT at an 80% sequence identity threshold [39], and each processed component dataset was then split into training and independent test sets at a 7:1 ratio.

Because MOSAIC is designed for cross-component transfer, we further audited potential sequence redundancy by calculating the maximum 41-bp center-aligned Hamming identity between each test sequence and training sequences from other component datasets. As summarized in Supplementary Table S2, highly similar cross-component matches were rare: only 24 of 129 728 test sequences (0.0185%) reached at least 90% identity, and only 310 sequences (0.2390%) reached the more relaxed 75% threshold. Moreover, a removal-based sensitivity analysis showed that excluding these rare cases did not materially change MOSAIC’s predictive performance or fairness metrics (Supplementary Table S2), indicating that the observed selective-transfer advantage is unlikely to be driven by sequence redundancy.

**Figure 1:**
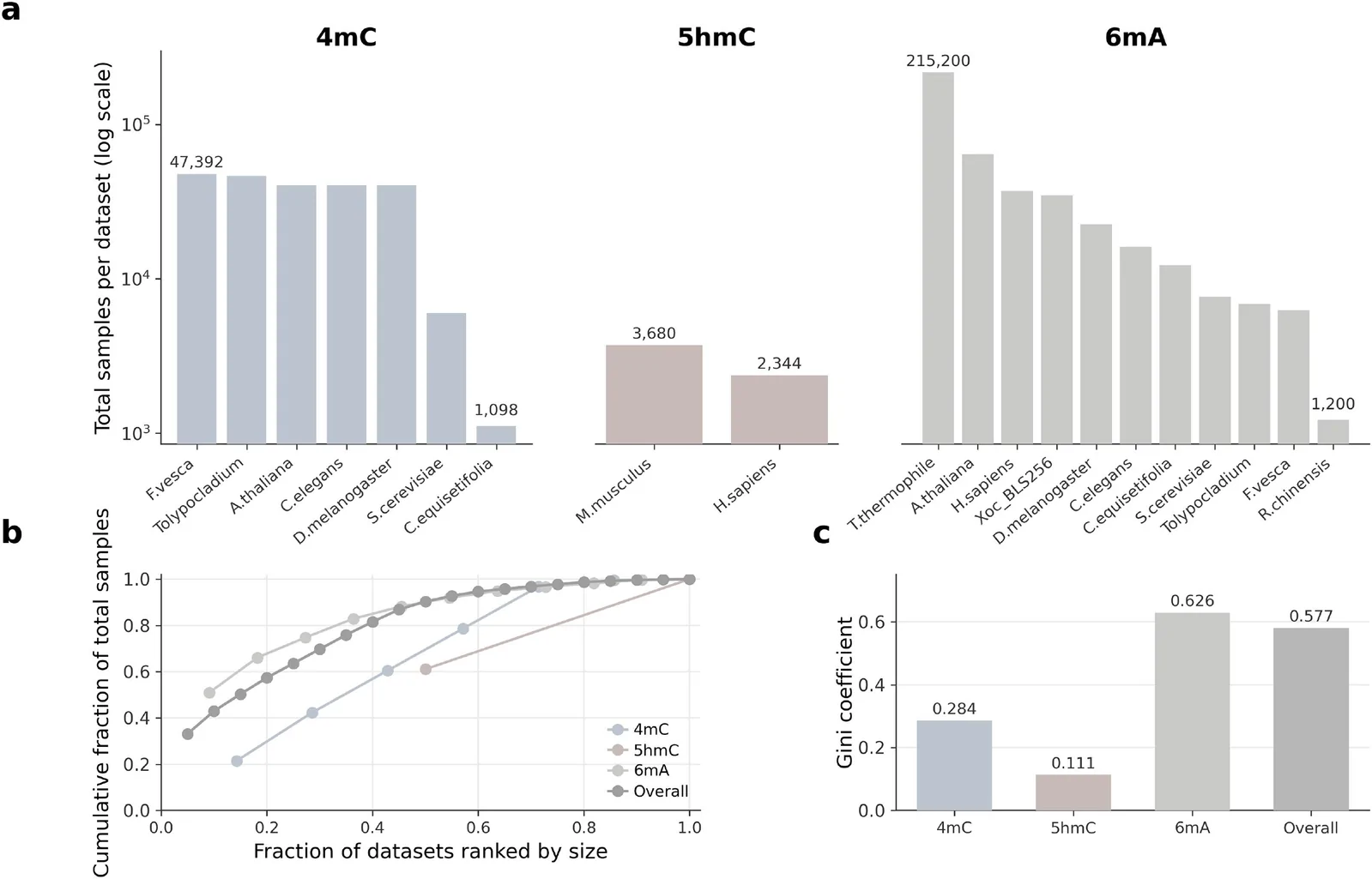
Domain imbalance across species and methylation types in the benchmark. (**a**) Total sample size of each component dataset in the 4mC, 5hmC, and 6mA groups. Although each component dataset is internally balanced at a 1:1 positive-to-unreported ratio, the benchmark as a whole remains strongly imbalanced across domains, with most samples contributed by a small number of high-resource datasets. (**b**) Cumulative fraction of total samples within each methylation type after ranking datasets by size. Each methylation group was independently normalized to a total of 1. Steeper curves indicate a greater concentration of samples in a small number of component datasets, consistent with a pronounced long-tailed distribution. (**c**) Gini coefficients of component dataset sizes for each methylation group. Higher values indicate stronger domain-level imbalance, with 6mA showing the greatest inequality in sample distribution.

A defining feature of this benchmark is its strong domain-level imbalance. Although each component dataset is internally balanced, the benchmark as a whole is highly uneven in scale, with sample sizes ranging from 1098 in the *Casuarina equisetifolia* 4mC dataset (4mC/*C. equisetifolia*) to 215 200 in the *Thermus thermophile* 6mA dataset (6mA/*T. thermophile*). As shown in Fig. 1a, a small number of high-resource domains contribute most of the available samples, whereas many other species remain low-resource. Figure 1b further highlights this pattern: within each methylation type, the cumulative sample curves rise steeply, showing that a limited number of component datasets account for a disproportionate share of the total samples. Figure 1c likewise shows that the degree of inequality varies across modification groups, with 6mA exhibiting the strongest domain-level imbalance, reflected in the highest Gini coefficient.

The benchmark also shows substantial heterogeneity across methylation types. The three modification groups differ in taxonomic composition, biological context, and sample scale. For example, in this collection, 5hmC is restricted to mammalian species, whereas 4mC and 6mA span a broader taxonomic range. Overall, the benchmark varies simultaneously in species background, modification type, and data volume, creating a central modeling challenge. Treating each component dataset as an independent task avoids direct cross-domain interference, but prevents systematic information sharing across related species and methylation types. Conversely, naive joint learning under such a long-tailed distribution is prone to biased optimization, with high-resource domains dominating parameter updates and the resulting model preferentially fitting majority domains. As a result, strong overall accuracy may coexist with systematically poorer performance in low-resource species or underrepresented modification groups, widening performance gaps across domains and reducing reliability in biologically less represented cases. Fair evaluation should therefore assess not only predictive accuracy, but also robustness across species and modification types under severe domain imbalance.

### Overall model architecture

**Figure 2:**
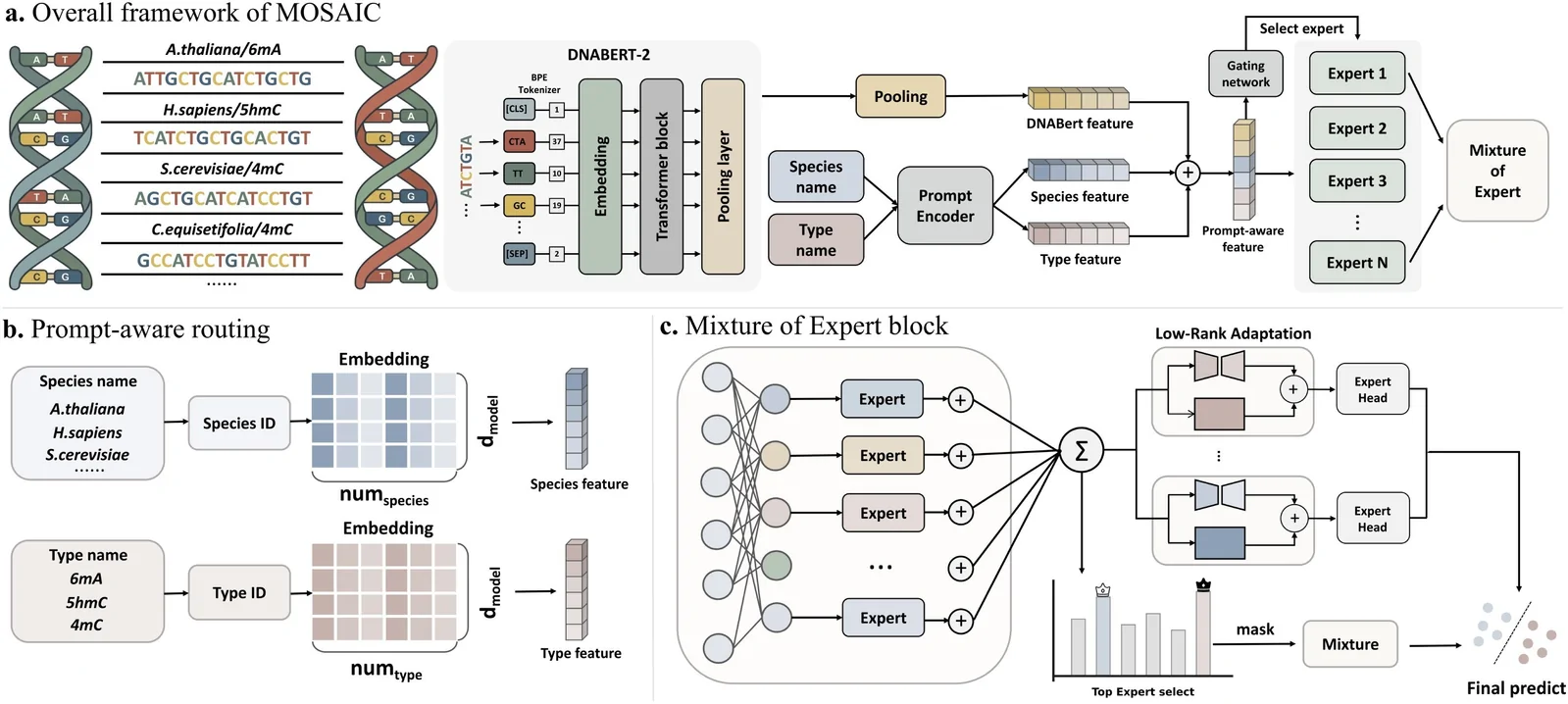
(**a**) Overall architecture of MOSAIC. Input DNA sequences from different species and methylation types are encoded by a shared DNABERT-2 backbone. In parallel, species and methylation-type prompts provide biological context, and the resulting prompt-aware features are combined with sequence features to guide expert selection. (**b**) Prompt-aware routing. Species identity and methylation type are mapped to learnable embeddings and transformed into species-specific and type-specific routing features. (**c**) Mixture-of-experts block. Prompt-aware routing activates a subset of experts, whose outputs are aggregated through a sparse mixture mechanism. Low-rank adaptation modules and expert-specific heads further promote specialization, and the fused representation is used for final prediction.

MOSAIC is a prompt-guided mixture-of-experts framework designed for cross-species and cross-modification methylation prediction under severe domain imbalance (Fig. 2). It combines a shared sequence encoder with biologically informed expert routing, allowing common sequence determinants to be learned jointly while retaining task-specific capacity for heterogeneous datasets.

Given an input DNA sequence window, MOSAIC first extracts sequence features using a shared DNABERT-2 backbone [30], while species identity and methylation type are encoded as learnable prompts to provide explicit biological context. These sequence and prompt features are then integrated and passed to a gating network for sparse expert selection. The expert pool contains 20 experts, 1 for each benchmark task; Supplementary Table S3 lists the correspondence among benchmark tasks, expert IDs, species prompts, methylation-type prompts, and dataset sizes, and Supplementary Table S4 summarizes the trainable model components and their training signals. Top-*k* routing with *k=4* is used to select one primary expert together with three auxiliary experts for each input. The primary expert is aligned with the input benchmark task through gating supervision, whereas the auxiliary experts provide controlled cross-task support. Their outputs are aggregated and passed to the final prediction head. By combining task-anchored primary routing with prompt-aware auxiliary routing, MOSAIC reduces cross-task interference under long-tailed domain imbalance while preserving the benefits of shared learning, thereby balancing task-specific specialization with selective information transfer across related methylation prediction tasks.

### DNABERT-2 backbone

DNABERT-2 serves as the shared sequence encoder in MOSAIC. As a genomic foundation model pretrained on large-scale multi-species DNA sequences, it provides a biologically informed representation space prior to task-specific optimization. This initialization is particularly valuable in the present setting, where methylation prediction must generalize across heterogeneous species and modification contexts. DNABERT-2 [30] was pretrained with masked language modeling on genome sequences from 135 species, comprising 32.49 billion nucleotides, and uses a byte-pair encoding (BPE) [40] vocabulary of size 4096.

For an input DNA sequence $x=(x_1,\dots ,x_L)$, BPE tokenization converts the nucleotide string into a variable-length token sequence


(1) \begin{eqnarray*}
x \rightarrow z=(z_1,\dots ,z_T), \qquad T \le L .
\end{eqnarray*}

Here, *z* denotes the BPE-tokenized representation of the original DNA sequence, $z_t$ is the *t*th token selected from the learned DNA BPE vocabulary, and *T* is the resulting token length after tokenization. The BPE vocabulary is learned by iteratively merging the most frequent adjacent symbol pair,


(2) \begin{eqnarray*}
(u^{*},v^{*})=\arg \max _{(u,v)} \mathrm{freq}(u,v), \qquad (u^{*},v^{*}) \rightarrow w ,
\end{eqnarray*}

where $\mathrm{freq}(u,v)$ denotes the corpus frequency of the pair *(u,v)* and *w* is the merged token. Compared with fixed *k*-mer tokenization, BPE yields variable-length tokens and shortens the effective input length.

After tokenization, each token is mapped to an embedding vector,


(3) \begin{eqnarray*}
\mathbf {h}_t^{(0)}=\mathbf {E}(z_t), \qquad t=1,\dots ,T ,
\end{eqnarray*}

and the resulting token embeddings are processed by a stack of Transformer encoder layers,


(4) \begin{eqnarray*}
\mathbf {H}^{(\ell )}=\mathrm{Transformer}^{(\ell )}\!\left(\mathbf {H}^{(\ell -1)}\right), \qquad \ell =1,\dots ,N ,
\end{eqnarray*}

where $\mathbf {H}^{(\ell )}=[\mathbf {h}_1^{(\ell )},\dots ,\mathbf {h}_T^{(\ell )}]$. A sequence-level representation is then obtained by [CLS] pooling,


(5) \begin{eqnarray*}
\mathbf {h}_{\mathrm{DNA}}=\mathbf {h}_{\mathrm{[CLS]}}^{(N)} .
\end{eqnarray*}

This vector serves as the shared sequence representation for downstream routing and prediction.

By initializing MOSAIC with a large-scale genomic pretrained model, the encoder starts from general sequence patterns and contextual dependencies learned across diverse genomes, rather than relying solely on task-specific training. Because the backbone is shared and optimized jointly across all tasks, it also provides a common feature space in which related species and methylation types can interact before selective expert routing is applied.

### Prompt-aware routing

Prompt-aware routing is the mechanism through which MOSAIC performs expert selection under cross-species and cross-modification heterogeneity (Fig. 2b). Rather than forcing all inputs through a uniformly shared pathway, MOSAIC conditions routing on explicit biological context, so that task-specific specialization can be preserved while related tasks can still exchange useful information [41, 42].

Biological context is encoded by two learnable embeddings, namely a species embedding and a methylation-type embedding,


(6) \begin{eqnarray*}
\mathbf {e}^{\mathrm{species}} = \mathbf {E}_{\mathrm{species}}(s),
\end{eqnarray*}

(7) \begin{eqnarray*}
\mathbf {e}^{\mathrm{type}} = \mathbf {E}_{\mathrm{type}}(m),
\end{eqnarray*}

where $\mathbf {e}^{\mathrm{species}}, \mathbf {e}^{\mathrm{type}} \in \mathbb {R}^{d}$, and *s* and *m* denote the species identity and methylation type, respectively.

These embeddings are added to the shared DNABERT-2 sequence representation to form the routing feature,


(8) \begin{eqnarray*}
\mathbf {h}^{\mathrm{route}} = \mathbf {h}^{\mathrm{DNA}} + \mathbf {e}^{\mathrm{species}} + \mathbf {e}^{\mathrm{type}}.
\end{eqnarray*}

The routing feature is then passed to a gating network to produce routing logits over the expert pool,


(9) \begin{eqnarray*}
\mathbf {a} = \mathbf {W}_{g}\mathbf {h}^{\mathrm{route}} + \mathbf {b}_{g},
\end{eqnarray*}

followed by


(10) \begin{eqnarray*}
\mathbf {p} = \mathrm{softmax}(\mathbf {a}),
\end{eqnarray*}

where $\mathbf {a} \in \mathbb {R}^{K}$ denotes the unnormalized routing-logit vector before softmax, $\mathbf {p} \in \mathbb {R}^{K}$ denotes the routing probabilities, and *K* is the number of experts.

Top-*k* routing is then applied to retain only the most relevant experts. For the selected expert set $\mathcal {S}=\mathrm{TopK}(\mathbf {p},k)$, the normalized sparse routing weights are defined as


(11) \begin{eqnarray*}
\tilde{p}_{i} = \left\lbrace \begin{array}{@{}l@{\quad }l@{}}\dfrac{p_i}{\sum \limits _{j \in \mathcal {S}} p_j}, & i \in \mathcal {S}, \\0, & i \notin \mathcal {S}. \end{array}\right.
\end{eqnarray*}

In MOSAIC, *K=20* and *k=4*, so the expert pool contains 20 experts and each input activates 4 of them. The choice of *k=4* was supported by an expanded hyperparameter sensitivity analysis across *k=1,… ,5*, with *k=4* providing the strongest overall balance across MCC, Avg-MCC, macro AUROC, macro AUPRC, and worst-dataset MCC (Supplementary Table S5). The highest-ranked expert serves as the task-specific primary expert and is aligned with the input benchmark task through the gating objective, whereas the remaining selected experts act as auxiliary experts. This design preserves a dedicated pathway for task-specific specialization while allowing complementary information to be drawn from other tasks when beneficial. Accordingly, Top-1, Top-2, Top-3, and Top-4 routing denote the first- through fourth-ranked experts in the selected set.

Prompt-aware routing therefore separates task anchoring from information transfer. The primary route preserves task identity, whereas the auxiliary routes provide controlled cross-task support under heterogeneous, long-tailed conditions.

### Mixture-of-experts with LoRA adaptation

Given the sparse routing weights produced by the prompt-aware router [31], the selected experts perform task-sensitive feature adaptation and prediction. This expert block adds specialized modeling capacity after routing, while remaining grounded in the shared DNABERT-2 representation.

For the *i*th expert, a lightweight LoRA-style residual adaptation is applied to the shared sequence feature $\mathbf {h}^{\mathrm{DNA}} \in \mathbb {R}^{d}$:


(12) \begin{eqnarray*}
\Delta \mathbf {h}_{i} = \frac{1}{r}\, \mathbf {W}^{\uparrow }_{i} \mathbf {W}^{\downarrow }_{i} \, \mathrm{Dropout}(\mathbf {h}^{\mathrm{DNA}}),
\end{eqnarray*}

where $\mathbf {W}^{\downarrow }_{i} \in \mathbb {R}^{r \times d}$, $\mathbf {W}^{\uparrow }_{i} \in \mathbb {R}^{d \times r}$, and *r* is the LoRA rank [43]. The adapted expert feature is then defined as


(13) \begin{eqnarray*}
\hat{\mathbf {h}}_{i} = \mathbf {h}^{\mathrm{DNA}} + \Delta \mathbf {h}_{i}.
\end{eqnarray*}

The expert adaptation is applied to $\mathbf {h}^{\mathrm{DNA}}$ rather than $\mathbf {h}^{\mathrm{route}}$ so that species and methylation-type prompts guide expert selection without being directly injected into the expert-specific sequence transformation. This separation keeps the adapted representation anchored in DNA sequence content while allowing task context to control routing. Each expert produces prediction logits through its own task-sensitive head,


(14) \begin{eqnarray*}
\mathbf {o}_{i} = \mathbf {W}^{(i)}_{c}\hat{\mathbf {h}}_{i} + \mathbf {b}^{(i)}_{c}, \qquad \mathbf {o}_{i} \in \mathbb {R}^{2},
\end{eqnarray*}

where $\mathbf {W}^{(i)}_{c}$ and $\mathbf {b}^{(i)}_{c}$ denote the parameters of the *i*th expert head. The final prediction logits are obtained by routing-weighted aggregation across experts,


(15) \begin{eqnarray*}
\mathbf {o} = \sum _{i=1}^{K} \tilde{p}_{i}\mathbf {o}_{i},
\end{eqnarray*}

followed by


(16) \begin{eqnarray*}
\hat{\mathbf {y}}=\mathrm{softmax}(\mathbf {o}).
\end{eqnarray*}

This design introduces expert-specific adaptation without replacing the shared backbone representation. The LoRA residual branch keeps the expert transformation lightweight, whereas the expert-specific heads enable task-sensitive prediction. Routing-weighted aggregation further allows multiple experts to contribute to the same sample, allowing specialization and auxiliary transfer to coexist within a single forward pass.

By combining shared sequence encoding with routed expert adaptation, the mixture-of-experts block preserves task-specific capacity while allowing complementary information to be drawn from related tasks under domain imbalance.

### Model optimization

Model training is jointly guided by routing supervision and final classification. The gating network is optimized with a multi-class cross-entropy loss [31],


(17) \begin{eqnarray*}
\mathcal {L}_{\mathrm{gate}} = -\sum _{i=1}^{K} y^{\mathrm{gate}}_{i}\log p_{i} = -\log p_{y^{\mathrm{gate}}},
\end{eqnarray*}

where *K* is the number of experts, $p_i$ is the routing probability assigned to the *i*th expert, and $y^{\mathrm{gate}}$ denotes the benchmark-task label of the input sample, represented as a one-hot target over the 20-expert pool. The final prediction is optimized with the standard classification loss,


(18) \begin{eqnarray*}
\mathcal {L}_{\mathrm{cls}} = -\sum _{c=1}^{C} y_{c}\log \hat{y}_{c},
\end{eqnarray*}

where *C* is the number of classes, $\hat{y}_{c}$ is the predicted probability for class *c*, and $y_c$ is the corresponding one-hot ground-truth label. The overall objective is defined as


(19) \begin{eqnarray*}
\mathcal {L} = \lambda _{\mathrm{cls}}\mathcal {L}_{\mathrm{cls}} + \lambda _{\mathrm{gate}}\mathcal {L}_{\mathrm{gate}},
\end{eqnarray*}

where $\lambda _{\mathrm{cls}}$ and $\lambda _{\mathrm{gate}}$ control the relative contributions of prediction and routing supervision, respectively. The loss weights were fixed during training rather than dynamically adjusted; the final setting used $\lambda _{\mathrm{cls}}=1.0$ and $\lambda _{\mathrm{gate}}=0.3$, selected from the sensitivity results in Supplementary Table S5. This formulation allows MOSAIC to optimize both task prediction and expert assignment in a unified manner [44].

### Rank-conditioned local sequence rule analysis

To interpret sequence-level signals associated with rank-specific expert dependence, we performed local sequence rule analysis on the aligned 41-bp windows centered on the candidate methylation site. Because all inputs were aligned to the same position, nucleotide preferences and short local patterns could be compared within a common coordinate system around the putative modified base [45].

Candidate local sequence rules were identified from the training set and independently evaluated on held-out test data [46]. At the dataset level, positive and unreported samples were compared using position-wise nucleotide enrichment, short *k*-mer enrichment (*k=3,4,5*), and summary local features, including GC content, CpG density, symmetry, palindrome score, and homopolymer length [47].

To relate these rules to expert dependence, a rank-conditioned matched-case design was used. Top-1 and Top-2 routing were analyzed separately, and only positive, correctly predicted samples were retained. For a given expert *e* and routing rank $r \in \lbrace 1,2\rbrace$, let $\mathcal {S}_{e,r}$ denote the set of samples in which expert *e* occupies rank *r*, and let $w_{i,e}^{(r)}$ denote the corresponding rank-specific routing weight for sample $x_i$. The high-dependence set was defined as


(20) \begin{eqnarray*}
E^{+}_{e,r} = \left\lbrace x_i \,\,\middle |\,\, x_i \in \mathcal {S}_{e,r},\,\, w_{i,e}^{(r)} \ge Q_{0.7}(\mathcal {S}_{e,r}) \right\rbrace ,
\end{eqnarray*}

where $Q_{0.7}(\mathcal {S}_{e,r})$ denotes the 70th percentile of the expert-specific routing-weight distribution at rank *r*. The robustness of this threshold choice is reported in Supplementary Table S6, where the 65th, 70th, and 75th percentile settings showed consistent pattern directions.

A matched control set $E^{-}_{e,r}$ was constructed from positive, correctly predicted samples from the same dataset with weaker dependence on expert *e* at rank *r*, defined as either


(21) \begin{eqnarray*}
\mathrm{Top}_{r}(x_j) \ne e,
\end{eqnarray*}

or


(22) \begin{eqnarray*}
w_{j,e}^{(r)} \le Q_{0.5}(\mathcal {S}_{e,r}),
\end{eqnarray*}

where $Q_{0.5}(\mathcal {S}_{e,r})$ denotes the expert-specific median routing weight at rank *r*. Matching was performed within each component dataset using nearest-neighbor matching on the predicted probability of the positive class, thereby minimizing differences in prediction confidence unrelated to expert dependence.

Position-wise sequence differences between $E^{+}_{e,r}$ and $E^{-}_{e,r}$ were summarized as differential sequence logos [48],


(23) \begin{eqnarray*}
\Delta f_{b,p} = f^{+}_{b,p} - f^{-}_{b,p},
\end{eqnarray*}

where $f^{+}_{b,p}$ and $f^{-}_{b,p}$ denote the frequencies of base *b* at position *p* in $E^{+}_{e,r}$ and $E^{-}_{e,r}$, respectively. Short local patterns were further evaluated by *k*-mer enrichment, and summary local features were compared between the two sets. Fisher’s exact test was used for *k*-mer enrichment, Mann–Whitney *U* tests were used for continuous local features [49–51], and multiple testing was controlled using the Benjamini–Hochberg procedure [52]. Candidate patterns were considered reproducible only if they showed consistent direction on held-out test data.

To assess whether auxiliary routing was preferentially amplified in low-resource datasets, the dataset-level mean Top-2 routing weight was defined as


(24) \begin{eqnarray*}
\bar{w}^{(2)}_{d} = \frac{1}{|\mathcal {D}^{+}_{d}|} \sum _{x_i \in \mathcal {D}^{+}_{d}} w_{i,\mathrm{Top}_{2}(x_i)}^{(2)},
\end{eqnarray*}

where $\mathcal {D}^{+}_{d}$ denotes the set of positive and correctly predicted samples from dataset *d*. The association between $\bar{w}^{(2)}_{d}$ and training-set size was then evaluated across datasets to determine whether auxiliary routing becomes more active under data scarcity [53].

### Zero-shot OOD evaluation

For zero-shot OOD evaluation, all external test sequences were held fixed and no fine-tuning on external data was performed. Prompt handling was prespecified according to the axis of distribution shift. Let $p_{+}(x\mid s,t)$ denote the model-predicted positive-class probability for sequence *x* under species prompt *s*, and methylation-type prompt *t*.

For target datasets in which 5mC was the unseen methylation type, the methylation-type prompt was fixed to 5hmC, the chemically closest methylation type represented during training. Let *s* denote the fixed, known species context of the target dataset. The positive-class probability for a target sequence *x* was therefore estimated as


(25) \begin{eqnarray*}
\hat{p}_{+}^{\mathrm{5mC}}(x;s) = p_{+}(x \mid s, \mathrm{5hmC}).
\end{eqnarray*}

For same-type, unseen-species datasets, let *t* denote the known methylation type of the target dataset, and let $\mathcal {P}_{t}$ be the set of $K_t$ species prompts represented during training for type *t*. The positive-class probability was estimated by uniformly averaging over these prompts:


(26) \begin{eqnarray*}
\hat{p}_{+}(x \mid t) = \frac{1}{K_t} \sum _{s \in \mathcal {P}_{t}} p_{+}(x \mid s,t).
\end{eqnarray*}

Target labels and target-set performance were not used to select a prompt or assign aggregation weights.

For the smallest external dataset (*n=252*), uncertainty in MCC was quantified using *B=20 000* class-stratified bootstrap resamples. Let $\mathcal {D}=\lbrace (y_i,\hat{y}_i)\rbrace _{i=1}^{n}$ denote the target-set labels and the corresponding fixed model predictions, and let $\mathcal {D}_{c}=\lbrace (y_i,\hat{y}_i)\in \mathcal {D}:y_i=c\rbrace$ be the subset with class label *c*, with $n_c=|\mathcal {D}_c|$. For bootstrap replicate *b*, label–prediction pairs were sampled with replacement within each class:


(27) \begin{eqnarray*}
\begin{aligned} \mathcal {D}_{c}^{(b)} &= \operatorname{SampleWithReplacement} \left(\mathcal {D}_{c},n_{c}\right), \quad c\in \lbrace 0,1\rbrace ,\\\mathcal {D}^{(b)} &= \operatorname{Concat} \left(\mathcal {D}_{0}^{(b)},\mathcal {D}_{1}^{(b)}\right), \quad b=1,\ldots ,B. \end{aligned}
\end{eqnarray*}

Writing $\mathrm{MCC}^{(b)}=\mathrm{MCC}(\mathcal {D}^{(b)})$, the 95% confidence interval was defined as


(28) \begin{eqnarray*}
\left[ Q_{0.025}\!\left(\lbrace \mathrm{MCC}^{(b)}\rbrace _{b=1}^{B}\right), \, Q_{0.975}\!\left(\lbrace \mathrm{MCC}^{(b)}\rbrace _{b=1}^{B}\right) \right],
\end{eqnarray*}

where $Q_q(\cdot )$ denotes the empirical *q*th quantile.

### Experimental setup and performance evaluation

Model performance was evaluated from two perspectives, namely predictive accuracy and cross-task fairness. Standard binary classification metrics included accuracy (ACC), precision (Prec), recall (Recall), F1-score (F1), Matthews correlation coefficient (MCC), area under the receiver operating characteristic curve (AUROC), and area under the precision-recall curve (AUPRC):


(29) \begin{eqnarray*}
\mathrm{ACC}=\frac{TP+TN}{TP+TN+FP+FN},
\end{eqnarray*}

(30) \begin{eqnarray*}
\mathrm{Prec}=\frac{TP}{TP+FP},
\end{eqnarray*}

(31) \begin{eqnarray*}
\mathrm{Recall}=\frac{TP}{TP+FN},
\end{eqnarray*}

(32) \begin{eqnarray*}
\mathrm{F1}=\frac{2\cdot \mathrm{Prec}\cdot \mathrm{Recall}}{\mathrm{Prec}+\mathrm{Recall}},
\end{eqnarray*}

(33) \begin{eqnarray*}
\mathrm{MCC}= \frac{TP\times TN-FP\times FN}{\sqrt{(TP+FP)(TP+FN)(TN+FP)(TN+FN)}},
\end{eqnarray*}

where *TP*, *TN*, *FP*, and *FN* denote the numbers of true positives, true negatives, false positives, and false negatives, respectively [54, 55]. AUROC is defined as the area under the receiver operating characteristic curve [56], whereas AUPRC summarizes the area under the precision-recall curve.

To quantify fairness under domain imbalance, fairness was defined as performance consistency across benchmark tasks rather than overall performance alone. Let $\mathcal {G}=\lbrace 1,\dots ,G\rbrace$ denote the set of benchmark tasks, where *G=20*, and let $m_g$ denote the MCC value of task *g*. Four fairness-related metrics were used. The average task performance was defined as


(34) \begin{eqnarray*}
\mathrm{Avg\mathrm{-}MCC}=\frac{1}{G}\sum _{g=1}^{G} m_g,
\end{eqnarray*}

which measures overall task-level performance. The worst-task MCC was defined as


(35) \begin{eqnarray*}
\mathrm{Worst\mathrm{-}MCC}=\min _{g\in \mathcal {G}} m_g,
\end{eqnarray*}

which reflects the performance lower bound across tasks and indicates whether underrepresented domains are systematically neglected [57].

To evaluate fairness with respect to resource imbalance, tasks were ranked by training-set size in descending order. The top 25% of tasks were defined as the head set $\mathcal {G}_{\mathrm{head}}$, and the bottom 25% were defined as the tail set $\mathcal {G}_{\mathrm{tail}}$. Since the benchmark contains 20 tasks, this corresponds to the 5 largest and 5 smallest tasks, respectively. The head–tail gap was then defined as


(36) \begin{eqnarray*}
\mathrm{Gap}_{\mathrm{head\mathrm{-}tail}} = \frac{1}{|\mathcal {G}_{\mathrm{head}}|}\sum _{g\in \mathcal {G}_{\mathrm{head}}} m_g - \frac{1}{|\mathcal {G}_{\mathrm{tail}}|}\sum _{g\in \mathcal {G}_{\mathrm{tail}}} m_g.
\end{eqnarray*}

A smaller $\mathrm{Gap}_{\mathrm{head\mathrm{-}tail}}$ value indicates a smaller performance disparity between high-resource and low-resource tasks [58].

Jain’s fairness index was further used to summarize performance equality across tasks:


(37) \begin{eqnarray*}
\mathrm{Jain} = \frac{\left(\sum _{g=1}^{G} m_g\right)^2}{G\sum _{g=1}^{G} m_g^2}.
\end{eqnarray*}

This index ranges from 0 to 1, with larger values indicating more balanced performance across tasks [59].

Together, these metrics assess whether a model not only achieves strong overall performance but also avoids systematic degradation in underrepresented tasks. Experiments were conducted using NVIDIA A100 40GB and NVIDIA RTX A6000 GPUs, and the final training hyperparameters are summarized in Supplementary Table S7.

## Results

The results are organized to evaluate selective transfer from complementary perspectives. We first compare MOSAIC with individual learning and conventional multi-task learning to test whether uniform sharing is sufficient for this benchmark. We then compare MOSAIC with existing methylation prediction tools under balanced and simulated imbalance settings. Subsequent ablation, model-control, and visualization analyses examine which architectural choices support the observed performance, whereas routing, perturbation, and OOD analyses assess how transfer is organized and how it behaves under external distribution shift. The final subsection describes the web server interface and output interpretation.

### Comparison with individual learning and traditional multi-task learning

Given the pronounced heterogeneity and head–tail imbalance of the 20 component benchmark, the first question is whether standard training strategies are sufficient or whether more selective cross-task transfer is needed. To address this question, MOSAIC is compared with two controlled baselines under the same model size, training time, and hardware settings. In *individual learning*, each task is trained independently using a separate DNABERT-based model. In *traditional multi-task learning*, all tasks share a single DNABERT backbone and differ only at the final classification stage. This comparison provides a direct assessment of three learning strategies: task-specific independent training, uniform parameter sharing, and selective sharing.

**Figure 3:**
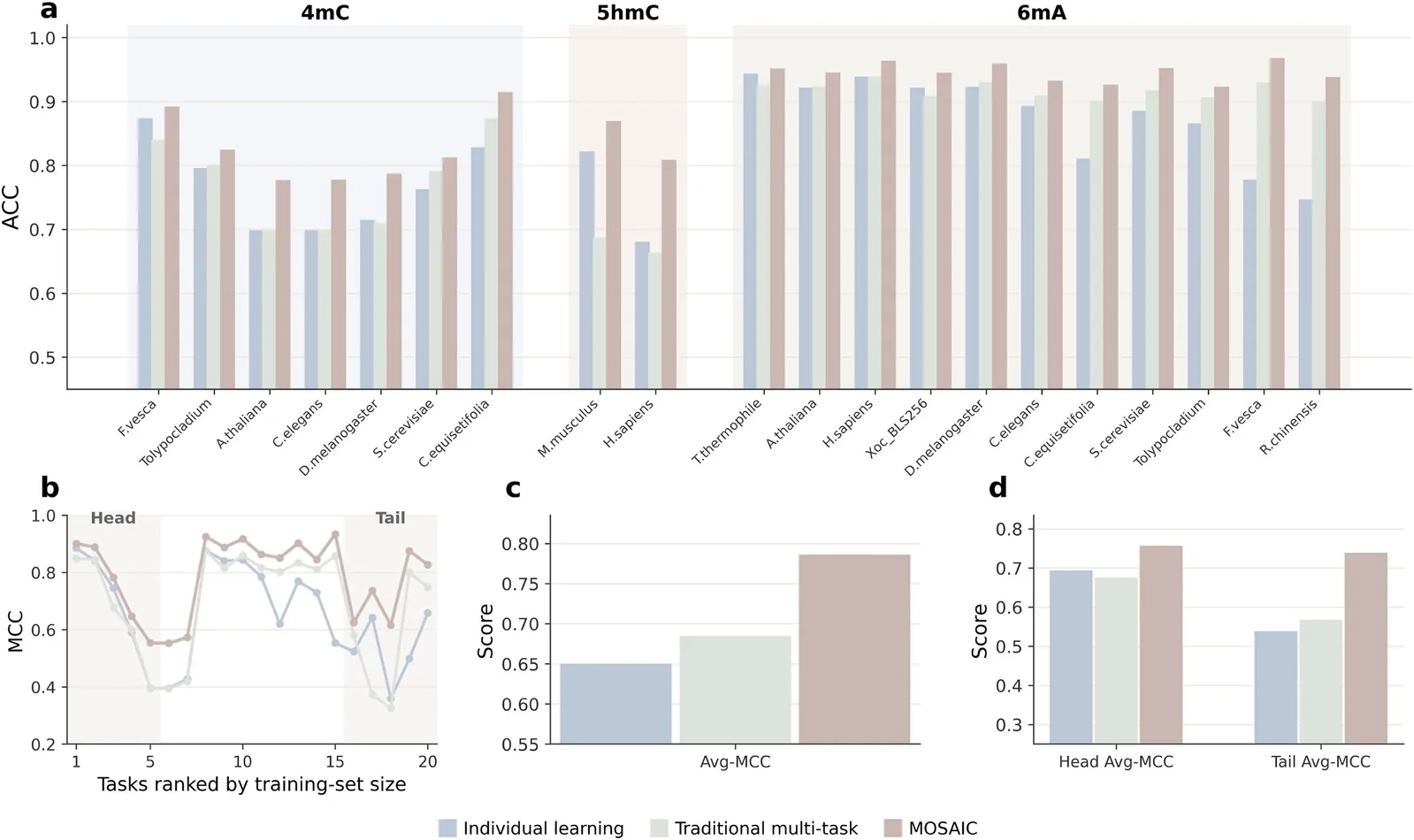
MOSAIC improves both predictive performance and cross-task fairness compared with individual learning and traditional multi-task learning. (**a**) Task-wise ACC across the 20-task benchmark, grouped by modification type (4mC, 5hmC, and 6mA). All three methods were evaluated under the same model size, training time, and hardware settings for a fair comparison. Relative to individual learning, traditional multi-task learning yields only modest, task-dependent gains and introduces clear performance trade-offs, with improvements on some tasks accompanied by declines on others. In contrast, MOSAIC delivers consistent ACC improvements across nearly the entire benchmark. (**b**) Task-wise MCC after sorting tasks by training-set size. Although traditional multi-task learning partially narrows the head–tail gap, its benefits remain uneven and several tail tasks still perform poorly. MOSAIC produces the most stable and uniformly improved task profile. (**c**) Avg-MCC across all tasks. (**d**) Avg-MCC for the head and tail subsets. MOSAIC improves performance in both subsets, with the largest gain observed in tail tasks, showing that its advantage extends beyond average accuracy to a substantially more balanced performance distribution across heterogeneous tasks. Head and tail tasks were defined as the top 25% and bottom 25% of benchmark tasks ranked by training-set size, respectively.

Figure 3a shows the accuracies for the 20 DNA methylation recognition tasks. Within each modification type (4mC, 5hmC, and 6mA), the tasks are ordered by decreasing component dataset size. Compared with individual learning, traditional multi-task learning improved performance on several tasks, confirming that shared optimization can recover useful information from other component datasets. However, the gains were uneven. Some tasks with relatively large training sets, such as 6mA/*T. thermophile* and 4mC/*F. vesca*, showed only modest improvement, whereas several low-resource tasks, including 4mC/*C. equisetifolia*, 6mA/*F. vesca*, and 6mA/*R. chinensis*, benefited substantially from cross-task transfer. Thus, performance did not vary monotonically with training set size. Although this may seem counterintuitive, the result highlights a key limitation of joint training: shared optimization can either improve or impair performance, depending on whether the transferred information is compatible with the biological context of the target task. Performance improved only when the transferred signal aligned well with the characteristics of the corresponding dataset.

In contrast, MOSAIC largely resolved this uneven performance. As shown in Fig. 3a, its improvements extended across nearly the entire benchmark rather than being confined to a small subset of favorable tasks. The same pattern is evident in Fig. 3 b–d, where MOSAIC achieved the highest Avg-MCC and the smallest head–tail gap. Moreover, even for the more difficult tasks in Fig. 3b, including ranks 5–7 and 16–18, where the absolute MCC values remained relatively low, MOSAIC still consistently outperformed both baselines. These results further support the view that the main limitation of conventional multi-task learning is not parameter sharing itself, but uncontrolled sharing across heterogeneous tasks, where knowledge transfer is not guided by task compatibility.

The fairness metrics further support this conclusion. As summarized in Table 1, traditional multi-task learning increased the average MCC from 0.649 to 0.684 and reduced the head–tail gap from 0.156 to 0.108 relative to individual learning. However, its worst MCC decreased from 0.359 to 0.326, and its fairness as measured by Jain’s fairness index likewise declined from 0.935 to 0.927. Thus, the improvement in average performance came at the cost of poorer performance on some tasks, indicating that the benefits of knowledge transfer were unevenly distributed. In contrast, MOSAIC achieved the highest average MCC (0.785), the highest worst MCC (0.553), the smallest head–tail gap (0.018), and the highest Jain’s fairness index (0.972), showing that its performance gains were distributed more evenly across the benchmark. Detailed task-wise comparisons and full metric summaries for these three training paradigms are provided in Supplementary Table S8.

**Table 1.** Performance and fairness comparison of different training paradigms

Model	Avg-MCC *↑*	Worst MCC *↑*	$\mathrm{Gap}_{\mathrm{head--tail}}$ *↓*	Jain index *↑*
Individual learning	0.649	0.359	0.156	0.935
Traditional multi-task	0.684	0.326	0.108	0.927
MOSAIC	**0.785**	**0.553**	**0.018**	**0.972**

*↑* indicates higher is better, whereas *↓* indicates lower is better.

### Comparison with existing methylation prediction tools

Benchmarking against existing methylation prediction tools further highlights the practical value of MOSAIC under both balanced and class-imbalanced evaluation settings. The comparison covers E-Net, MSNet, SNNRice, UniMethylNet, iDNA-OpenPrompt, and Methyl-GP. To maintain comparability, methods that required model training were trained on the same benchmark splits whenever applicable, and all methods were evaluated using the same test sequences and metric definitions.

**Figure 4:**
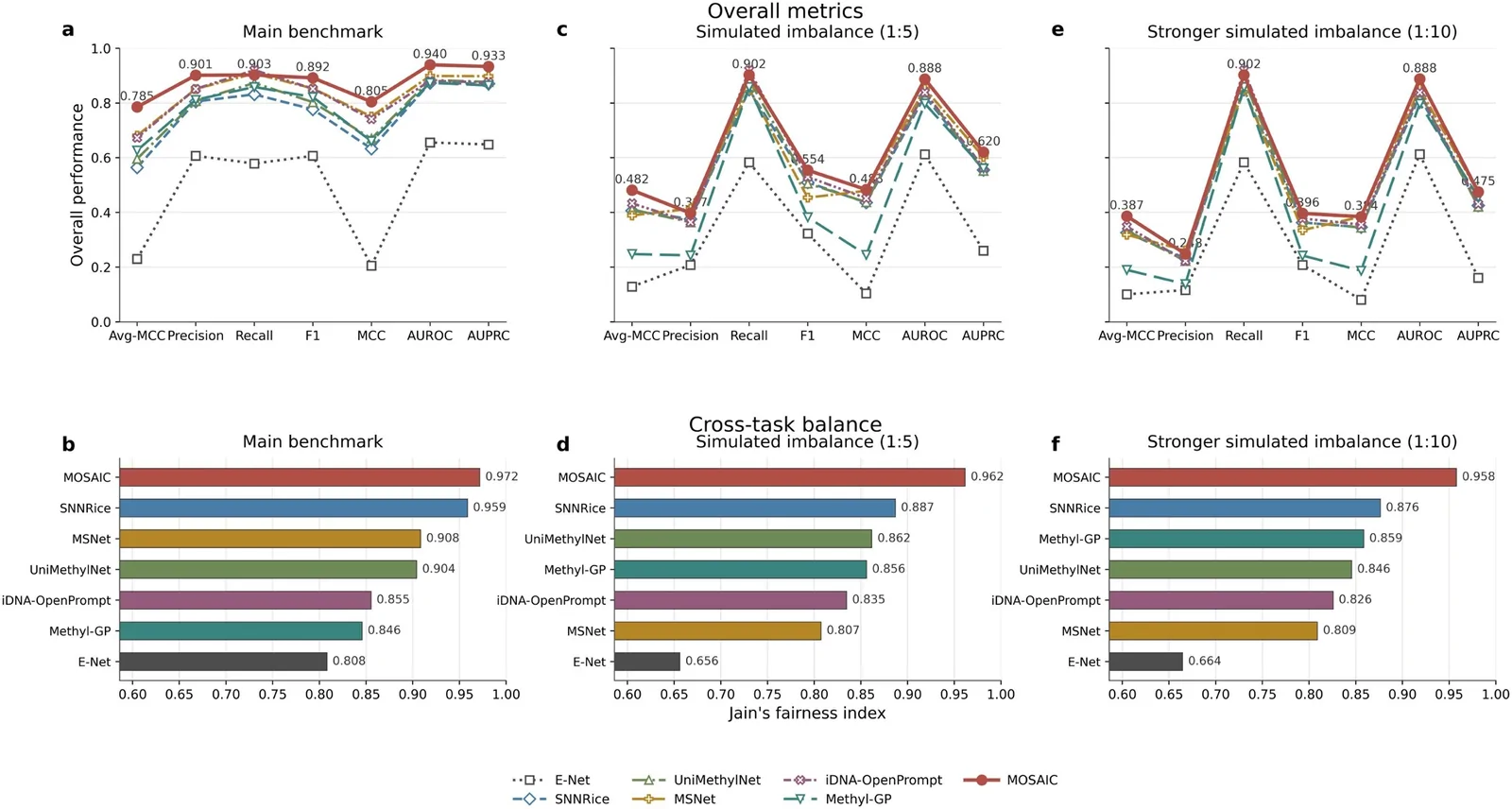
Comparison of MOSAIC with existing methylation prediction tools under balanced and simulated imbalance settings. (**a, c, e**) Overall performance profiles on the main balanced benchmark, the simulated 1:5 imbalance test set, and the simulated 1:10 imbalance test set, respectively. Displayed metrics include Avg-MCC, precision, recall, F1-score, MCC, AUROC, and AUPRC. (**b, d, f**) Cross-task balance measured by Jain’s fairness index under the corresponding settings. MOSAIC maintains the strongest overall fairness across all three settings, while remaining competitive across threshold-dependent and ranking-based predictive metrics.

On the main balanced benchmark, MOSAIC showed the strongest overall profile among the compared methods (Fig. 4a). It achieved the highest Avg-MCC (0.785), MCC (0.805), AUROC (0.940), and AUPRC (0.933), while also maintaining high precision, recall, and F1-score. Thus, the advantage of MOSAIC was not confined to a single threshold-dependent metric, but was also reflected in ranking-based evaluation. This predictive gain was accompanied by the strongest cross-task balance (Fig. 4b), with a Jain’s fairness index of 0.972 compared with 0.959 for the next highest method. These results indicate that MOSAIC improves benchmark-level prediction while keeping per-dataset performance more evenly distributed across heterogeneous tasks.

The simulated imbalance evaluations further tested whether this behavior persisted when the positive-to-unreported ratio was shifted by adding same-species central-base genome-candidate sequences treated as unreported for methylation. Because ACC becomes less informative under shifted class ratios, the imbalance panels emphasize Avg-MCC, MCC, AUROC, AUPRC, and Jain’s fairness index (Fig. 4c–f). Under the 1:5 setting, MOSAIC achieved the highest Avg-MCC (0.482), MCC (0.483), AUROC (0.888), AUPRC (0.620), and Jain’s fairness index (0.962). Under the stronger 1:10 setting, MOSAIC again achieved the highest Avg-MCC (0.387), MCC (0.384), AUROC (0.888), AUPRC (0.475), and Jain’s fairness index (0.958). These results show that the advantage of MOSAIC under simulated sparse candidate-site conditions was not limited to threshold-dependent classification metrics, but also extended to ranking-based evaluation and cross-dataset balance.

The preservation of high cross-dataset fairness under simulated imbalance is particularly relevant to the selective-transfer goal of MOSAIC. On the main balanced benchmark, MOSAIC achieved a Jain’s fairness index of 0.972, compared with 0.959 for the strongest non-MOSAIC method. Under the 1:5 and 1:10 imbalance settings, MOSAIC maintained high Jain’s fairness indices of 0.962 and 0.958, whereas the strongest non-MOSAIC values were 0.887 and 0.876, respectively. Consequently, the fairness gap increased from 0.013 on the main benchmark to 0.075 under 1:5 and 0.082 under 1:10. This widening gap suggests that MOSAIC did not gain performance by shifting toward easier or higher-resource component datasets, but retained comparatively stable support across more difficult datasets under sparse candidate-site conditions. Overall, MOSAIC retained a strong and balanced profile when the evaluation distribution became more sparse. The complete benchmark method comparison and the 1:5 / 1:10 imbalance summaries are reported in Supplementary Table S9.

To further cover predictors developed for individual methylation types, representative single-modification models were also evaluated, including MultiScale-CNN-4mCPred for 4mC, MEDCNN-5hmC for 5hmC, and p6mA for 6mA. Because these tools are not unified cross-task models, they were compared only on the methylation types for which they were designed and were not included in the cross-task fairness analysis. MOSAIC remained competitive in this matched comparison, with higher Avg-MCC across the 20 corresponding dataset-level comparisons (0.785 versus 0.713) and higher MCC in 15 of 20 datasets. The complete dataset-level results are listed in Supplementary Table S10.

### Structural ablation and model-control analyses of MOSAIC

To identify which architectural components drove the performance of MOSAIC, a structural ablation study was first conducted by removing or replacing key modules in the model architecture, including expert specialization, biologically informed routing, sparse expert selection, and local task-specific refinement. These variants were designed to test architecture-level contributions, whereas additional model-control and hyperparameter analyses were reported separately in the Supplementary Tables. All structural variants were evaluated on the same benchmark, with identical data splits, optimization schedules, and evaluation protocols, to ensure a fair comparison.

Specifically, "w/o MoE" removes the entire mixture-of-experts block, reducing the model to a fully shared backbone with a single prediction head. This variant is therefore similar to the traditional multi-task learning baseline based on standard shared optimization. "w/o Prompt Routing" removes biologically informed expert allocation, together with the supervision signal that stabilizes the dominant expert pathway. As a result, experts are selected without access to biological context or task identity. *Full Soft Routing* retains the full expert pool but replaces sparse top-*k* selection with dense weighted aggregation, so that all experts contribute to each prediction. Although their contributions remain weighted by routing probabilities, and some may be very small, no expert is completely excluded. This variant therefore represents a dense mixture-of-experts model rather than the sparse formulation used in MOSAIC. "w/o Adapter" removes the lightweight post-backbone refinement module and thus tests the role of local task-specific feature adjustment after routing. Finally, "w/o MoE + Adapter" removes both expert specialization and post-backbone refinement, yielding a largely shared model with only limited task sensitivity. This variant is again similar to the traditional multi-task learning baseline. Together, these architecture-level variants assess the contributions of expert specialization, biologically informed routing, sparse expert selection, and local adaptation within the full MOSAIC architecture.

**Figure 5:**
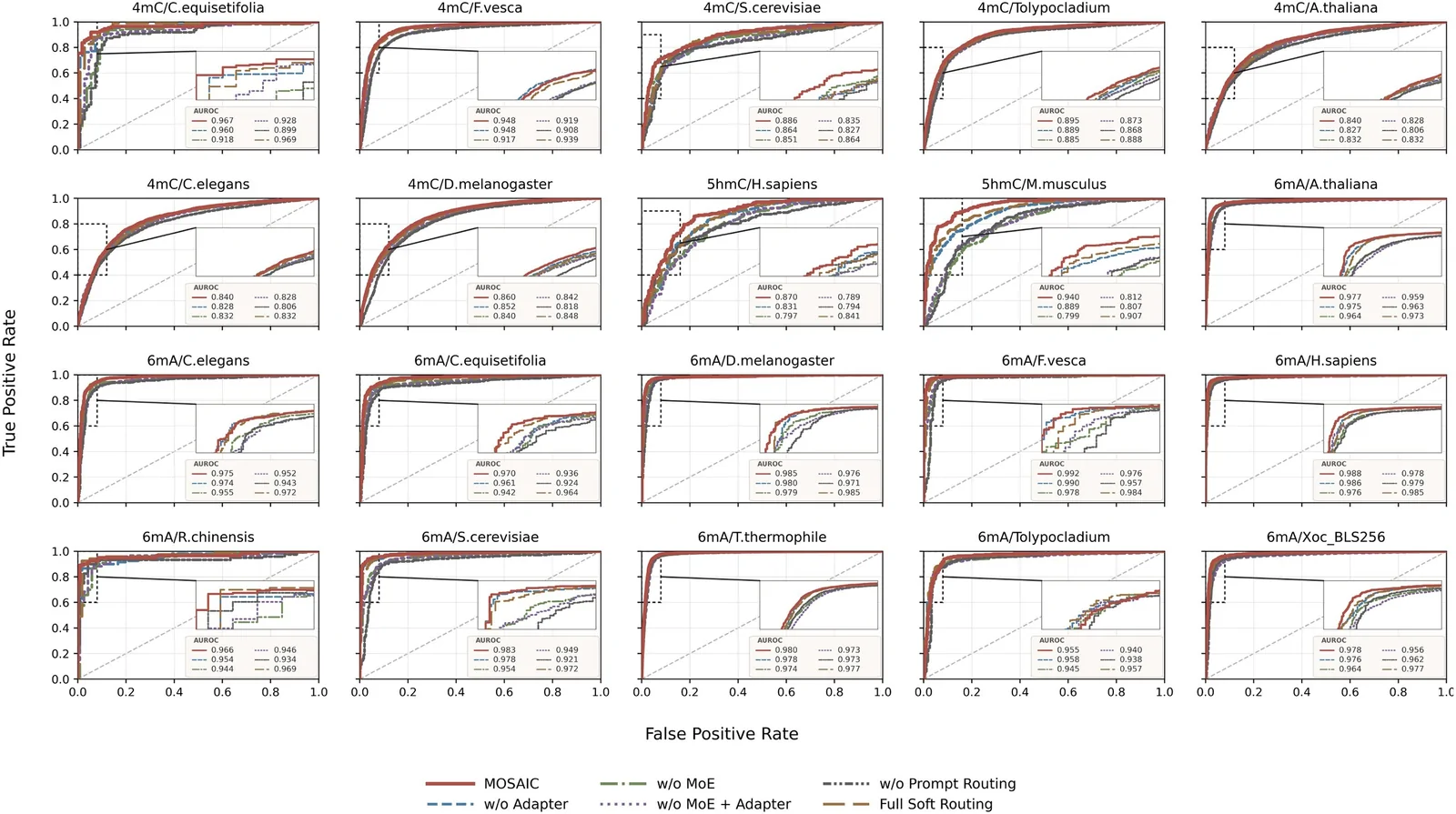
ROC comparison of MOSAIC and five ablated variants across all 20 component datasets. Each panel shows the dataset-specific ROC curves together with the corresponding AUROC values. Dashed rectangles indicate the low-FPR regions shown at higher magnification in the insets, with connecting lines linking each inset to its source region. Across tasks spanning 4mC, 5hmC, and 6mA, MOSAIC achieves the best ROC profile in most cases, indicating that its advantage is broadly distributed rather than driven by only a few benchmark tasks. The largest performance declines are observed after removing the MoE module or prompt-aware routing, highlighting the importance of expert specialization and context-aware expert allocation. The *Full Soft Routing* variant shows an intermediate loss, consistent with reduced expert selectivity under dense aggregation.

ROC curves for all 20 tasks are shown in Fig. 5. As expected, the full model, MOSAIC, generally achieves the strongest ROC profiles across the benchmark, whereas the ablated variants show similar or poorer performance. The largest declines were observed for "w/o MoE", "w/o Prompt Routing", and "w/o MoE + Adapter" on 5hmC/*M. musculus*, 5hmC/*H. sapiens*, 4mC/*C. equisetifolia*, 6mA/*R. chinensis*, and 6mA/*S. cerevisiae*. These results indicate that accurate expert selection is critical for discrimination performance and depends on the joint presence of both the mixture-of-experts module and prompt-aware routing. Removing the mixture-of-experts block reduces the model to a less selective shared representation, whereas removing prompt-aware routing disrupts expert recruitment based on biological context. The resulting ROC degradation is especially pronounced in low-resource tasks, highlighting the importance of sparse, context-aware expert selection. Interestingly, "Full Soft Routing" remained relatively competitive on several high-resource datasets, but its performance deteriorated on low-resource datasets, such as 5hmC/*M. musculus* and 5hmC/*H. sapiens*. This pattern suggests that allowing access to all experts is not always advantageous, particularly when task-relevant specialization must be preserved under long-tailed imbalance. In practical terms, when training data are insufficient to support effective learning across the full expert pool, relying on one specialized expert together with a small number of auxiliary experts can lead to better decisions under limited data conditions.

**Figure 6:**
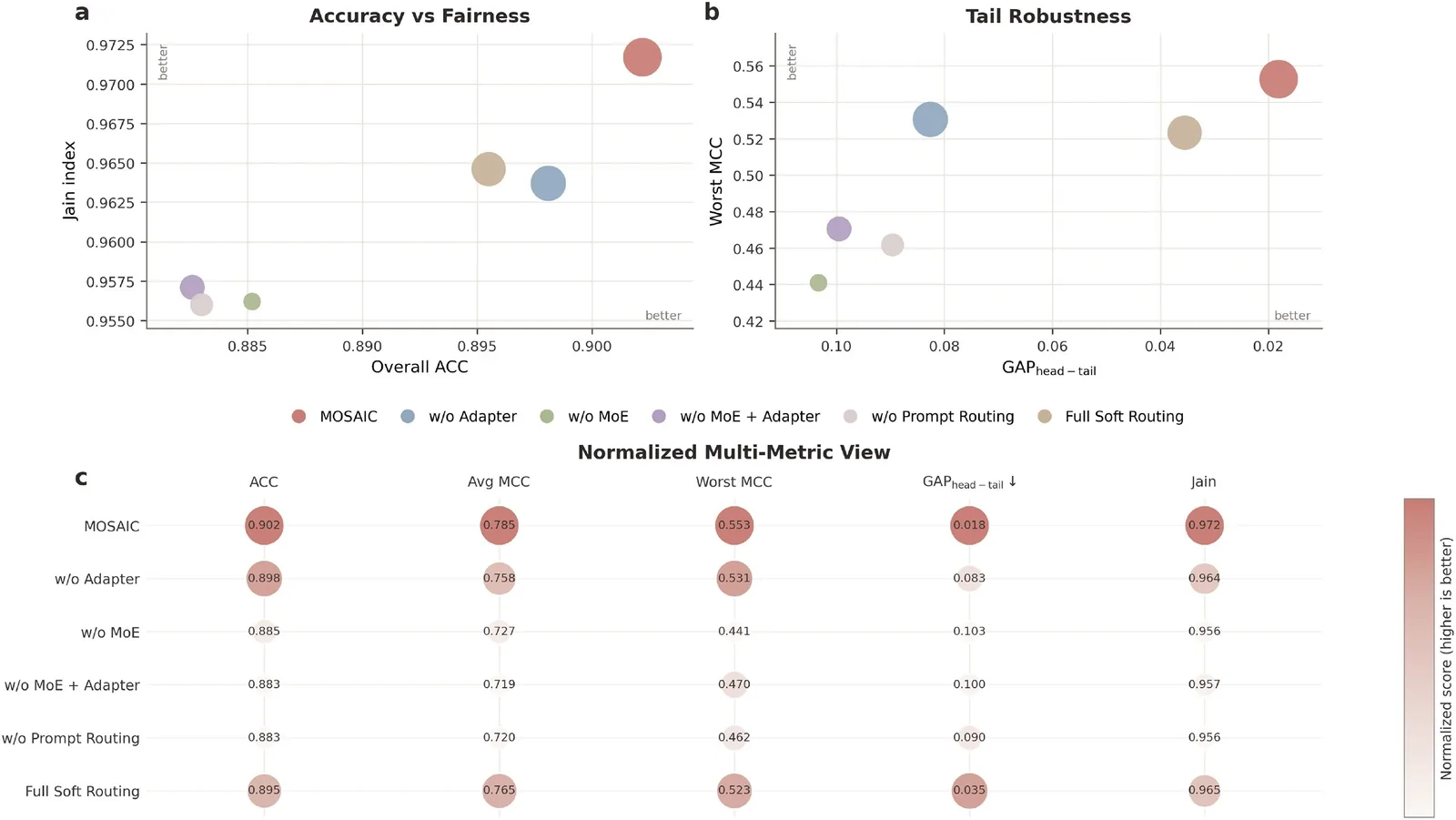
Ablation analysis of MOSAIC from the perspectives of predictive performance and cross-dataset fairness. (**a**) Joint comparison of ACC and Jain’s fairness index, with bubble size proportional to worst MCC. MOSAIC occupies the most favorable accuracy-fairness region, indicating that its performance gains are achieved without sacrificing balance across heterogeneous datasets. (**b**) Tail robustness assessed by the head–tail gap and worst MCC. MOSAIC shows the smallest $\mathrm{Gap}_{\mathrm{head\!-\!tail}}$ together with the highest worst-case performance, demonstrating superior robustness on underrepresented datasets. Note that the $\mathrm{Gap}_{\mathrm{head\!-\!tail}}$ decreases from left to right. (**c**) Normalized multi-metric summary across ACC, average MCC, worst MCC, $\mathrm{Gap}_{\mathrm{head\!-\!tail}}$, and Jain’s fairness index. MOSAIC shows the best overall performance, whereas removing prompt-aware routing, the MoE block, or sparse expert selection reduces predictive performance, fairness, or both.

The performance differences become clearer when fairness across datasets is taken into account. As shown in Fig. 6a, which plots overall accuracy against Jain’s fairness index, MOSAIC occupies the upper-right corner, indicating the strongest combination of predictive performance and cross-dataset balance. By contrast, *w/o MoE, w/o Prompt Routing*, and *w/o MoE + Adapter* shift toward the lower-left region, consistent with their weaker ROC performance. This fairness-oriented view also helps resolve variants with similar average performance. Although *w/o Adapter* and *Full Soft Routing* remain close in overall accuracy and Jain’s fairness index, they separate more clearly in Fig. 6b, where head–tail gap is plotted against worst MCC. Relative to *Full Soft Routing, w/o Adapter* shows a larger head–tail gap, indicating poorer balance across heterogeneous datasets despite similar average performance. In contrast, *Full Soft Routing* retains tail robustness closer to that of MOSAIC, suggesting that preserving the expert structure alone already provides some benefit. Overall, the inferior worst-task performance and larger head–tail disparity observed in *w/o MoE, w/o Prompt Routing*, and *w/o MoE + Adapter* indicate that both the mixture-of-experts design and biologically guided routing are important for maintaining balanced performance, particularly on low-resource tasks.

Finally, a normalized multi-metric summary, including accuracy, average MCC, worst MCC, head–tail gap, and Jain’s fairness index, is presented in Fig. 6c. MOSAIC again achieves the strongest overall performance, whereas each ablation weakens at least one critical dimension of performance. Taken together, these results demonstrate that the advantage of MOSAIC does not arise from one single architectural component, but from the coordinated contributions of expert specialization, biologically guided routing, sparse expert selection, and local refinement. Detailed numerical results for all structural ablation variants are provided in Supplementary Table S11. These structural ablation results are complemented by model-control analyses. A prompt-tuning variant was also evaluated under the same benchmark setting as an additional comparison for lightweight adaptation strategies (Supplementary Table S12). This comparison reflects the functional difference between the two adaptation designs. Prompt Tuning mainly adjusts learnable prompt embeddings to steer the shared representation, whereas LoRA modifies the expert-specific feature transformation after sparse routing with a small number of trainable parameters. The stronger performance of the LoRA-based MOSAIC variant should therefore be interpreted as supporting the adopted expert-adaptation design in this framework, rather than as an exhaustive single-factor comparison of all possible Prompt Tuning implementations. For the backbone comparison, both a from-scratch Mamba backbone [60] and a DNA-pretrained Mamba backbone [61] were evaluated. DNA pretraining substantially improved the Mamba-based variant relative to training from scratch, but Mamba-based backbones still did not improve performance over the DNABERT-2-based MOSAIC (Supplementary Table S13). Prompt and routing-control results are summarized in Supplementary Table S14 and Supplementary Fig. S4. Randomizing the species prompt, methylation-type prompt or both prompts during inference substantially reduced performance, and a sample-wise random gating-label control also reduced Avg-MCC and worst-dataset MCC relative to the correct-prompt setting. These results indicate that both prompt context and meaningful routing supervision contribute to the selective-transfer behavior of MOSAIC.

### Feature-space visualization of expert-adapted representations

**Figure 7:**
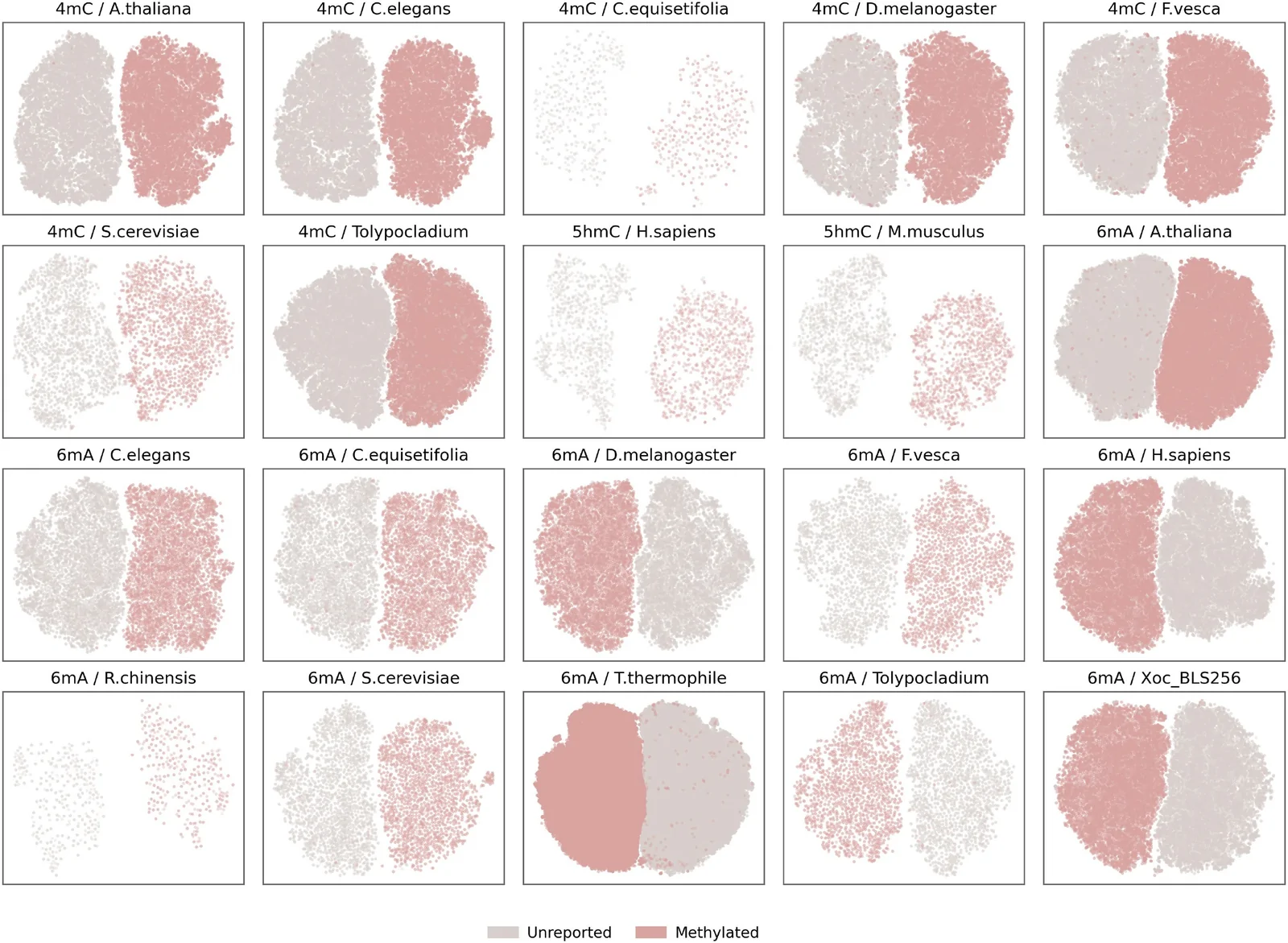
t-SNE visualization of expert-adapted feature representations learned by MOSAIC across 20 methylation datasets spanning 4mC, 5hmC, and 6mA tasks. Each panel corresponds to one dataset, and points are colored by class label (methylated or unreported). Across diverse species and methylation types, MOSAIC organizes the learned representations into visually apparent class-associated structure.

The distribution of samples in the latent space induced by the expert-adapted feature representations of MOSAIC was further examined. For visualization, the latent representations were projected into two dimensions using t-SNE, and the results for all 20 datasets are shown in Fig. 7. The t-SNE projection was generated with a perplexity of 30, automatic learning-rate selection, and random seed 42. Across the benchmark, methylated and unreported samples generally formed visually separable groups. These groups tended to be denser in high-resource datasets and sparser in low-resource datasets. Because t-SNE is a qualitative nonlinear projection, these patterns are best interpreted as a visual summary of the learned representation space rather than as direct evidence of stable decision boundaries. Nevertheless, the visualization is consistent with the design of MOSAIC, in which shared sequence modeling is combined with expert-specific adaptation, allowing transferable structure to be retained while sharpening task-relevant representations.

### Hierarchical expert routing and its local sequence basis

**Figure 8:**
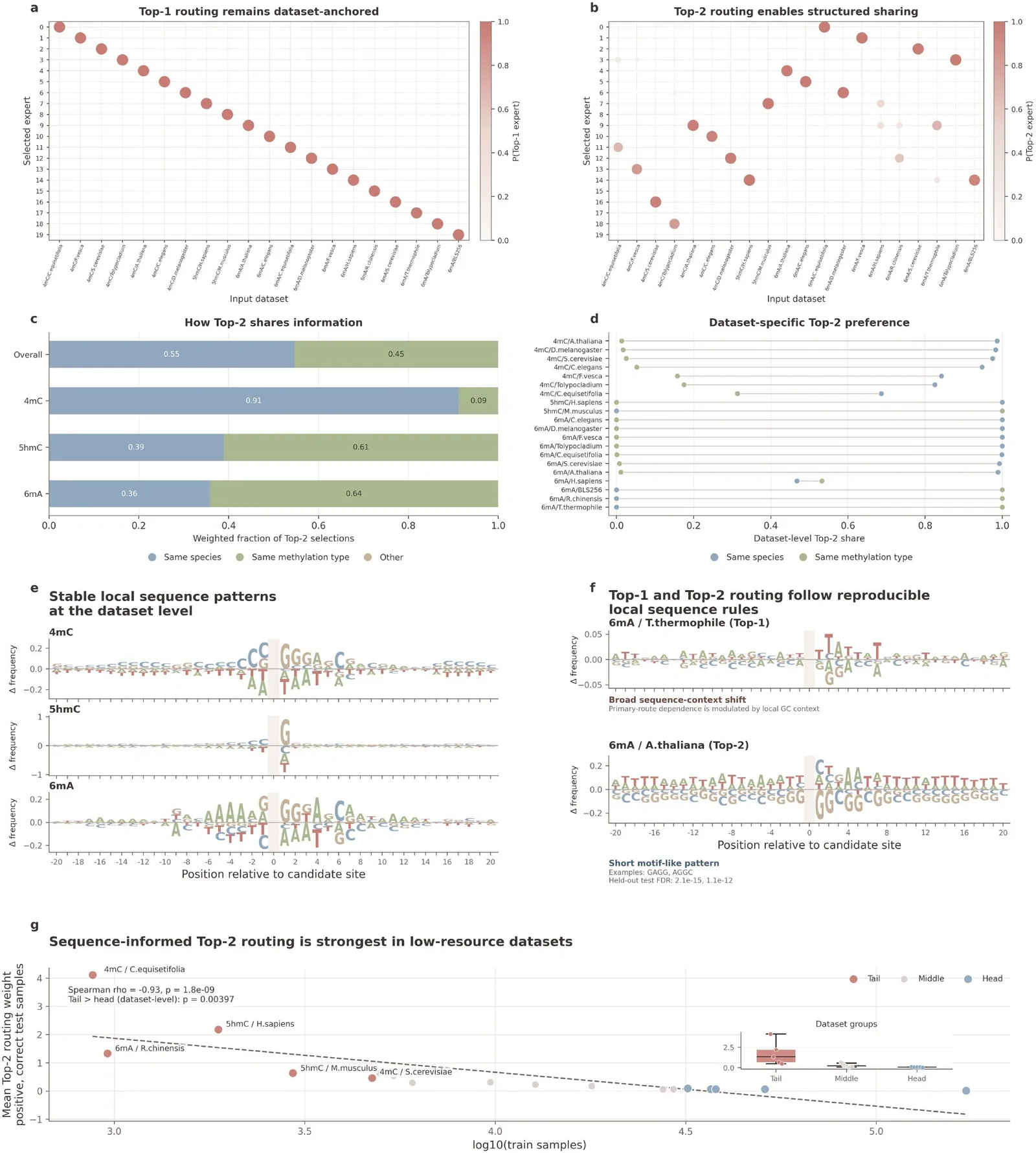
Hierarchical expert routing and its local sequence basis in MOSAIC. (**a**) Top-1 routing for each input dataset. The selected expert indices follow the same order as the input datasets. For example, 0 denotes 4mC/*C. equisetifolia*. Top-1 routing is exclusively dataset-anchored, indicating that the dominant route preserves task-specific specialization. (**b**) Top-2 routing for each input dataset. Top-2 routing introduces structured auxiliary sharing, in some cases involving multiple component datasets. (**c**) Aggregate Top-2 sharing patterns reveal that auxiliary experts are selected primarily on the basis of either species similarity or methylation-type similarity. (**d**) Dataset-level Top-2 preferences further show that the dominant sharing pattern varies across tasks. (**e**) Differential sequence logos comparing positive and unreported samples across methylation types indicate that the aligned 41-bp window contains methylation-related local sequence patterns. (**f**) Representative rank-conditioned differential logos for a Top-1 primary expert and a Top-2 auxiliary expert show that local sequence patterns modulate dependence on rank-specific experts under prompt-aware routing. (**g**) Mean Top-2 routing weight across datasets. The weight is highest in low-resource datasets and lowest in high-resource datasets, suggesting an asymmetric, sequence-informed transfer mechanism in which auxiliary experts preferentially support tail datasets while limiting harmful reverse transfer into well-supported head datasets.

Previous analyses showed that context-aware sparse expert selection is critical to the performance of MOSAIC, raising the question of which experts are actually recruited during this process. Figure 8a shows the Top-1 experts selected for each component dataset. For every task, the Top-1 expert is always the corresponding task-specific expert, indicating a dominant dataset-anchored routing pattern. This primary assignment preserves task-specific structure and gives MOSAIC a behavior analogous to individual learning at the first routing level. Once this primary anchoring is established, Top-2 routing begins to recruit the first auxiliary expert from outside the target task. The Top-2 routing results are shown in Fig. 8b. Unlike Top-1 routing, Top-2 expert selection can vary across input samples from the same task, so multiple experts may be recruited as Top-2 experts with different probabilities. To clarify these patterns, we further grouped auxiliary experts into three categories: those from the same species but a different methylation type (termed “same species” hereafter), those from the same methylation type but a different species (termed “same methylation type” hereafter), and those from different species and different methylation types (termed “other” hereafter). Aggregating the results in Fig. 8b in this way yields the summary shown in Fig. 8c. As shown in Fig. 8c, Top-2 experts are recruited exclusively from either the same species or the same methylation type. Moreover, the recruitment preference differs across methylation types: for 4mC, Top-2 experts are recruited predominantly from the same species, whereas for 5hmC and 6mA they are more often recruited from the same methylation type. This pattern is biologically plausible. The strong same-species preference in 4mC aligns with the view that 4mC-associated sequence context is more lineage-dependent and more tightly coupled to species-specific genomic background [62, 63]. In contrast, the preference for the same methylation type in 5hmC and 6mA is consistent with the idea that these marks share more transferable local sequence features across related biological contexts [63–66]. A detailed breakdown of Top-2 routing preferences for each component dataset can be found in Fig. 8d.

To determine whether these routing preferences were associated with specific biological sequences, we first applied local motif-like rule analysis to the sample sequences in each methylation group. The results are presented in Fig. 8e. Because all samples were 41 bp long and centered on the candidate site (C or A), local nucleotide preferences could be compared directly in a common positional framework, with larger letters indicating higher occurrence probabilities. Methylation-associated local sequence patterns can therefore be inspected directly from the figure. For example, methylated 4mC and 5hmC sites often showed a CCCG-like pattern, while 6mA sites tended to exhibit a GAGG-like pattern. By contrast, unreported sites displayed patterns such as AACT, TTCT, CAAA, etc. These task-dependent nucleotide preferences suggest that methylation-associated sequence signals are primarily localized within a short sequence context near the candidate site, rather than distributed across a broader region [64].

Similarly, for each component dataset, we analyzed the relationship between sequence patterns and expert routing using a conservative rank-conditioned design (see the “Materials and methods” section for details). Candidate patterns were identified from the training set and independently evaluated on the held-out test set. Top-1 and Top-2 routing were analyzed separately. For a given expert and routing rank, $E^{+}$ denotes correctly predicted positive samples in which that expert occupies the specified rank and exhibits strong rank-specific dependence, whereas $E^{-}$ denotes matched positive controls from the same dataset with weaker dependence on the same expert. By matching within each dataset and restricting the analysis to correctly predicted positive samples, this design controls for prompt context, class label, and overall dataset composition. The resulting sequence patterns reflect signals that modulate expert behavior. Fig. 8f shows the strongest associations, defined by the lowest held-out test FDR, between sequence patterns and Top-1 or Top-2 routing. Unlike the patterns in Fig. 8e, the Top-1 example does not show a dominant local motif. Instead, two broad, non-local sequences are observed: one with a positive *Δ* frequency, which increases the relevance of the Top-1 expert in 6mA/*T. thermophile* recognition, and the other that decreases its contribution. In comparison, the Top-2 example shows a short motif-like pattern, GAGG, which reduces the relevance of the associated auxiliary expert. This result indicates that auxiliary expert recruitment is associated with sequence patterns consistent with methylation-related local context rather than reflecting an arbitrary secondary ranking. Corresponding plots for all component datasets and for both Top-1 and Top-2 routing are provided in Supplementary Fig. S1.

Finally, the importance of Top-2 routing was assessed across the 20 component datasets (Fig. 8g). In held-out test samples, the mean Top-2 routing weight showed a strong negative association with training-set size (Spearman *ρ =-0.93*, $p=1.8\times 10^{-9}$), indicating that tail datasets relied more heavily on Top-2 routing than head datasets (*P=.00397*). This pattern suggests that auxiliary routing is recruited asymmetrically according to data availability. When a component dataset is too small to support full learning of all relevant sequence patterns, MOSAIC preferentially recruits auxiliary experts to incorporate motif-related information from related species or methylation types, thereby improving predictive performance. By contrast, head datasets remain much less dependent on Top-2 routing, indicating that transfer is not imposed uniformly across tasks but introduced primarily where it is needed. Such asymmetry is advantageous because it allows low-resource datasets to benefit from useful local sequence information while limiting harmful reverse transfer into well-supported head datasets. Analogous Top-3 and Top-4 auxiliary routing maps and their relation compositions are shown in Supplementary Fig. S2.

### Further analysis of auxiliary routing

**Figure 9:**
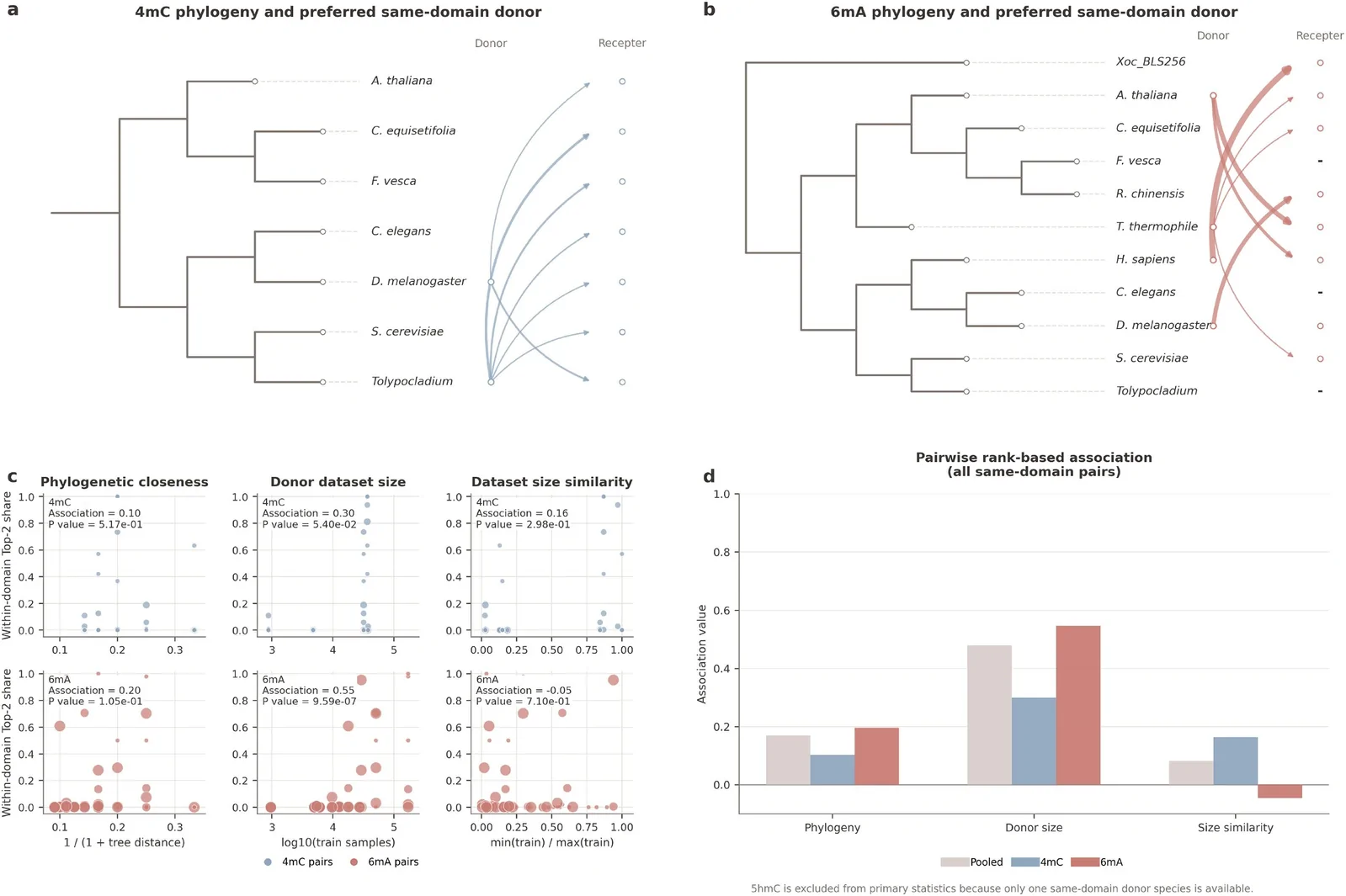
Phylogenetic proximity versus dataset-related factors in same-domain Top-2 routing. (**a**) Schematic phylogeny of the species represented in the 4mC datasets, annotated with the preferred major donors. Arrows indicate the flow of information, and arrow thickness reflects the frequency of requests. (**b**) Corresponding analysis for 6mA. Branch lengths represent relative phylogenetic proximity rather than calibrated divergence time. (**c**) Pairwise comparison of within-domain Top-2 sharing with three candidate explanatory factors: donor–acceptor phylogenetic proximity, donor dataset size, and donor–acceptor dataset-size similarity. The upper row shows results for 4mC, and the lower row shows results for 6mA. In each subplot, the association value denotes the Spearman rank correlation between the predictor and within-domain Top-2 sharing, and the accompanying *P*-value corresponds to a two-sided test of zero association. **(d)** Summary of the pairwise rank-based associations for the same three predictors. Pooled denotes the association recalculated after combining all eligible same-domain donor–acceptor pairs from 4mC and 6mA into a single analysis set, rather than averaging the two domain-specific values. Across both domains, donor dataset size shows the strongest association with Top-2 preference, whereas phylogenetic proximity is weaker and dataset-size similarity has only a limited effect.

After establishing that Top-2 auxiliary routing is sequence-informed, we next asked which source dataset provides the most relevant sequence information. From the perspective of homology modeling, one might expect sequence features learned from phylogenetically closer species to be more informative. In contrast, data-driven learning often suggests that features derived from high-resource datasets are more influential. To address this question, we analyzed the preferred Top-2 donors at both the dataset and pairwise levels. As already shown in Fig. 8c, 4mC methylation prediction predominantly recruits Top-2 experts from the same species but with different methylation types, indicating that phylogenetic relatedness plays an important role in information sharing. For the more limited sharing within the same 4mC methylation type, the preferred donors and their phylogenetic relationships are shown in Fig. 9a. The panel illustrates the major information flows from donor species to acceptor species, where major donors are defined by the frequency with which they are recruited by acceptor species. Arrow thickness reflects the size of the acceptor dataset. The major donors are *Tolypocladium* and *Drosophila melanogaster*, both of which are high-resource species. A similar pattern is observed for 6mA datasets (Fig. 9b), which shows the preferred information flow within the same domain. The major donors are *T. thermophile, Arabidopsis thaliana, Homo sapiens*, and *D. melanogaster*. That is, four of the five largest 6mA datasets. Interestingly, although *Xoc_BLS256* is the fourth-largest 6mA dataset, it is not selected by any acceptor species during Top-2 expert recruitment. This observation is consistent with the fact that *Xoc_BLS256* is phylogenetically isolated from the other 6mA species, suggesting that its sequence information is less useful than that of other high-resource but more closely related species. Nevertheless, *Xoc_BLS256* itself frequently recruits Top-2 experts from *H. sapiens*, as indicated by the thick arrow. Again, the thicker arrow reflects only the large size of the *Xoc_BLS256* dataset and does not imply that this information sharing is especially relevant or critical for model performance. To further characterize donor-to-acceptor information flow, that is, how Top-2 experts are recruited, we plotted the association value, which is defined as the normalized ratio of Top-2 expert recruitment for each acceptor species, in Fig. 9c. The results are organized according to donor–acceptor phylogenetic proximity, donor dataset size, and donor-acceptor dataset-size similarity. Consistent with the observations above, donor dataset size remains the strongest predictor of Top-2 knowledge sharing for both 4mC and 6mA, as shown in the middle panels. However, the phylogenetic analysis for 6mA (bottom left) also indicates that sharing between evolutionarily close species does occur, whereas sharing from the phylogenetically isolated species (*Xoc_BLS256*) is essentially absent. Dataset-size similarity can also be informative in some cases, particularly for 4mC (top right panel). At the domain level, Fig. 9d shows a similar trend. Donor dataset size is again the primary factor of a major donor, whereas phylogenetic proximity plays a secondary but still detectable role in auxiliary expert routing. Together, these results suggest that under the current model architecture and dataset scale, phylogenetic information is partially captured by the model: Top-2 experts are generally recruited from higher-resource datasets, unless the corresponding species is phylogenetically isolated. Analogous phylogeny-based analyses for Top-3 and Top-4 auxiliary routing are shown in Supplementary Fig. S3.

**Figure 10:**
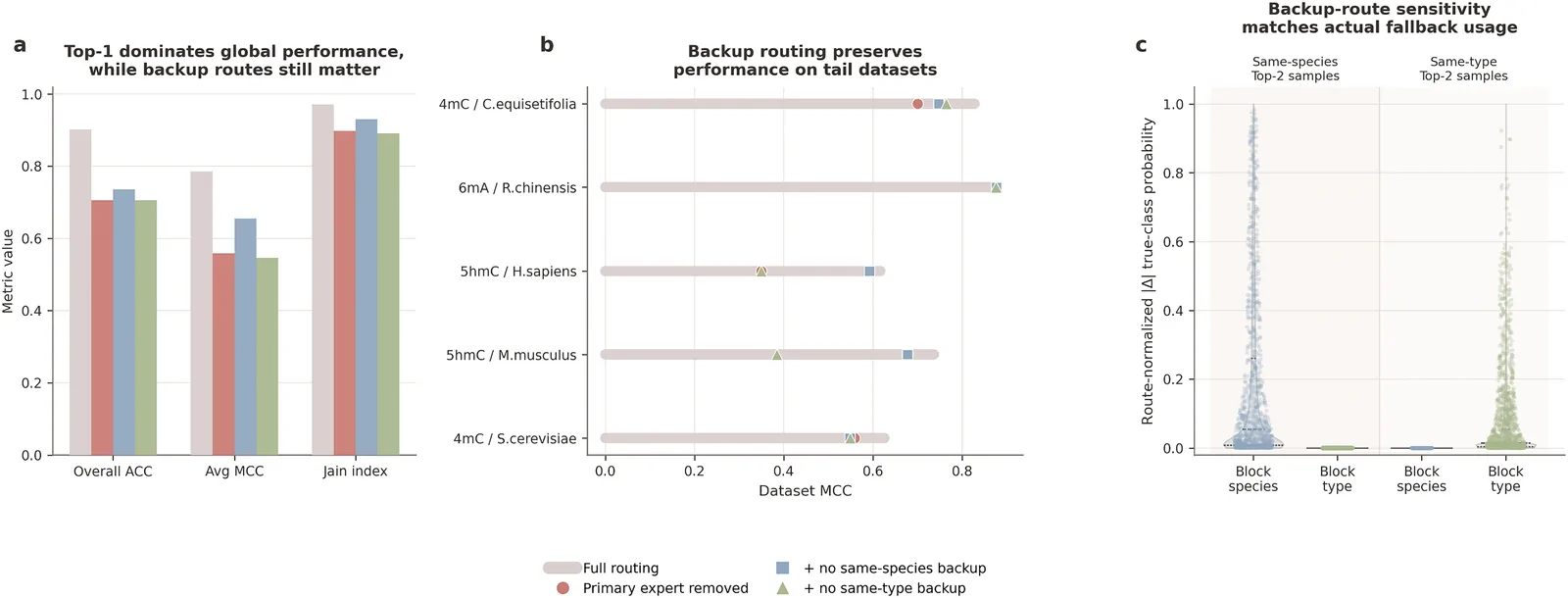
Perturbation analysis of dominant and backup routing in MOSAIC. (**a**) Overall benchmark performance under full routing, removal of the primary expert, and additional blocking of same-species or same-type backup routes after primary-expert removal. Metrics shown are overall ACC, average MCC, and Jain’s fairness index. (**b**) MCC on representative tail datasets under the same perturbations, illustrating heterogeneous dependence on fallback routing across datasets. (**c**) Sample-level sensitivity to backup-route blocking. Samples are grouped according to whether their selected Top-2 donor is from the same species or the same methylation type, and the y-axis shows the absolute change in true-class probability induced by blocking the indicated backup class, normalized within each route group. Sensitivity is concentrated on the backup relation actually used, with larger shifts observed for same-species Top-2 samples.

Finally, perturbation analysis was used to examine how MOSAIC responds when the dominant (Top-1) and auxiliary routing paths are selectively disrupted (Fig. 10). The exact intervention definitions, including whether prompts were kept fixed, whether routing logits were recomputed, and whether remaining weights were renormalized, are summarized in Supplementary Table S4. At the benchmark level, removal of the primary expert produced the largest overall decline (Fig. 10a), with overall ACC decreasing from 0.902 to 0.706, average MCC from 0.785 to 0.559, and Jain’s fairness index from 0.972 to 0.897. These drops indicate that the dominant route carries most of the model’s predictive strength and much of its balanced performance across tasks. At the same time, performance was not abolished after primary-expert removal, showing that auxiliary experts still retain non-trivial predictive information. Once the primary expert was removed, further blocking either same-species or same-type auxiliary routes produced only modest additional changes at the benchmark level, suggesting that these backup routes provide residual support but do not compensate for the loss of the dominant route globally.

Greater heterogeneity emerged at the dataset level. Fig. 10b shows that tail datasets differed markedly in their sensitivity to routing perturbation. For example, 5hmC/*H. sapiens* exhibited a pronounced MCC decrease after removal of the primary expert, whereas 6mA/*R. chinensis* remained largely stable under the same perturbation. Other datasets showed selective sensitivity to particular backup disruptions. These results indicate that dependence on fallback routing is strongly dataset-dependent rather than uniform across the benchmark, and that aggregate trends can obscure substantial variation among individual low-resource datasets.

The basis of this heterogeneity is further clarified by Fig. 10c. In this panel, samples were grouped according to whether the selected Top-2 donor belonged to the same species or to the same methylation type, and the route-normalized absolute change in true-class probability was measured after blocking the corresponding auxiliary route. When the Top-2 donor originated from the same species, blocking same-species auxiliary routes induced the largest probability shifts, particularly in species-dominated datasets, which were mainly 4mC tasks. This pattern is consistent with the earlier observation that Top-2 auxiliary experts in these datasets are preferentially recruited from the same species. In contrast, samples whose Top-2 donor came from the same methylation type showed a weaker and more concentrated response near zero, indicating that same-type auxiliary routing provides a more limited residual contribution on average. Taken together, these results show that auxiliary routing in MOSAIC does not function as generic reserve capacity. Instead, perturbation has the strongest effect when it targets the backup route actually used by the model, with same-species auxiliary routing serving as the more influential residual channel in this benchmark.

### Zero-shot OOD evaluation reveals structured transfer under external distribution shift

**Figure 11:**
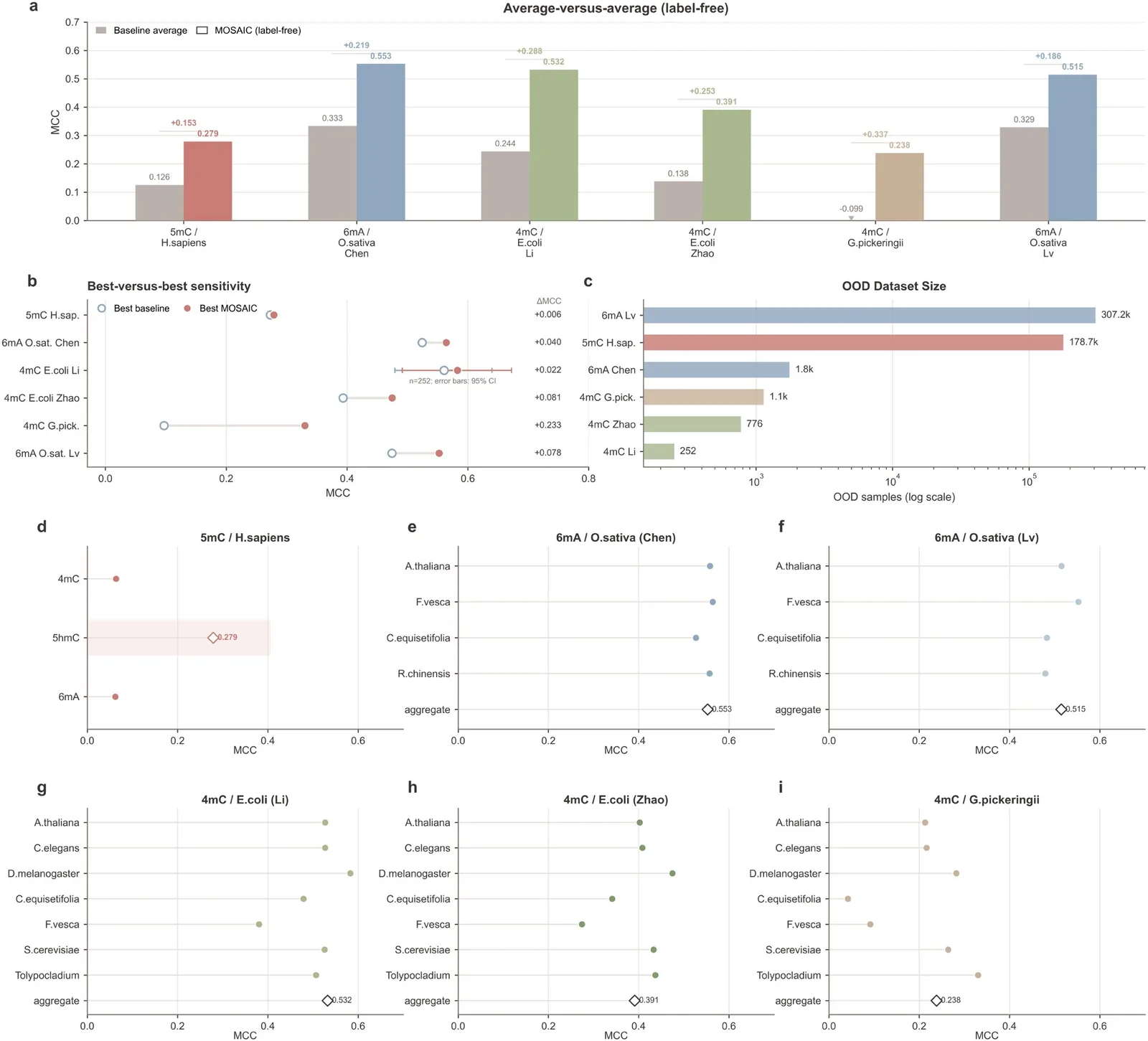
Zero-shot OOD evaluation across six fully separated external datasets. The datasets comprise one same-species, unseen-methylation-type setting and five same-methylation-type, unseen-species settings. No external-data fine-tuning was performed. For 5mC in *H. sapiens*, the *H. sapiens* context was fixed and 5hmC was the prespecified single surrogate prompt. For unseen-species datasets, positive-class probabilities were uniformly averaged over all seen species prompts for the fixed methylation type; target labels and target-set performance were not used to select a prompt or averaging weight. (**a**) Primary average-versus-average MCC comparison. Gray bars denote the mean of the evaluated public baselines and colored bars denote MOSAIC under the label-free rule. (**b**) Matched best-versus-best comparison on the same six datasets. The open marker denotes the best individual baseline and the filled marker denotes the best observed MOSAIC prompt result within the fixed candidate sweep. Error bars for the 252-sample 4mC/*E. coli* dataset denote 95% class-stratified bootstrap confidence intervals from 20 000 resamples. This panel applies the same best-result summary to MOSAIC and the baselines and complements the label-free primary comparison in panel (a). (**c**) OOD sample sizes. (**d**–**i**) Prompt-level MCC profiles. For 5mC in *H. sapiens*, the diamond marks the fixed 5hmC surrogate score. For the 6mA and 4mC unseen-species datasets, the diamond marks the score from uniform mean-probability aggregation across the same-type prompt set. Full OOD metrics are reported in Supplementary Table S15.

To test whether MOSAIC generalizes beyond the original random-split benchmark, zero-shot OOD evaluation was performed on six fully separated external datasets, including 5mC in *H. sapiens* [67], two 6mA datasets in *O. sativa* [33, 68], and three 4mC bacterial datasets from species not used for MOSAIC training [13]. These datasets represent one unseen-type setting and five unseen-species settings. The external sequences were fixed, no external-data fine-tuning was performed, and the primary MOSAIC estimates followed the prespecified label-free prompt rules described above. Together, these datasets contained 489 827 OOD samples, exceeding the 129 728 samples in the original benchmark test set, with per-dataset sizes ranging from 252 to 307 236 samples (Fig. 11c).

Figure 11a summarizes the matched average-versus-average MCC comparison under no target-data prior information. MOSAIC under the label-free rule exceeded the mean of the evaluated public baselines on all six datasets: 0.279 versus 0.126 on 5mC in *H. sapiens*; 0.553 versus 0.333 and 0.515 versus 0.329 on the two 6mA *O. sativa* datasets; and 0.532 versus 0.244, 0.391 versus 0.138, and 0.238 versus −0.099 on the three 4mC bacterial datasets. Figure 11 b provides the matched best-versus-best comparison after evaluation of the fixed candidate sets. The best observed MOSAIC MCC within each fixed prompt sweep exceeded the best individual baseline on all six datasets, with margins ranging from 0.006 to 0.233. Thus, panel (b) applies the same best-result summary to MOSAIC and the baselines and complements the label-free primary estimate in panel (a). For the smallest 4mC/*E. coli* dataset (*n=252*), the primary label-free MCC was 0.532 (95% CI, 0.439–0.623), whereas the best observed MOSAIC MCC was 0.583 (95% CI, 0.491–0.672), compared with 0.561 (95% CI, 0.479–0.640) for the best individual baseline; the overlapping intervals warrant cautious interpretation of this dataset in isolation.

Prompt-level profiles further clarify how the prespecified rules operate (Fig. 11d–i). In the unseen-type setting, the fixed 5hmC surrogate prompt yielded an MCC of 0.279 for 5mC in *H. sapiens*. In the unseen-species settings, the reported score was calculated from the uniform mean of positive-class probabilities across the same-type seen-species prompts. The two 6mA *O. sativa* datasets reached aggregate MCC values of 0.553 and 0.515, while the three 4mC bacterial datasets reached 0.532, 0.391, and 0.238. Supplementary Fig. S5m retains the corresponding multi-metric comparison, with positive MCC gains from +0.153 to +0.337, AUROC gains from +0.121 to +0.250, and AUPRC gains from +0.049 to +0.162. The complete dataset-level OOD metrics, including sample counts, MCC, AUROC, and AUPRC, are reported in Supplementary Table S15. The supplementary prompt and routing profiles show that dominant Top-2 fallback routes were prompt-dependent rather than diffuse, consistent with selective transfer within the available seen contexts. Together, these results support a measured conclusion that MOSAIC shows a degree of zero-shot transfer within the evaluated OOD settings, rather than a general claim of unrestricted generalization.

### Web server deployment

To facilitate the practical use of MOSAIC, we developed a dedicated web server and made it publicly available at https://ycclab.cuhk.edu.cn/MOSAIC/index.php. The server provides an interactive interface for methylation prediction and model interpretation, allowing users to specify the target methylation type and corresponding species prompt before submitting query sequences for analysis. In addition to short, model-compatible inputs, the server also supports full-length DNA sequences, which are automatically segmented into 41-bp windows using a sliding-window procedure for user convenience.

Beyond prediction alone, the server explicitly exposes the expert selection process during inference. Along with the prediction results, the interface displays the routed experts selected for each query, including the primary expert and auxiliary experts. A typical output therefore includes the predicted methylation label, the output score, the selected primary and auxiliary experts, and their routing weights under the user-specified species and methylation-type prompts. This allows users to inspect how a given methylation type and species prompt are mapped to expert modules and how additional experts are recruited to provide structured auxiliary support. By linking prediction outputs to the internal routing process, the server offers a direct and accessible view of the prompt-aware routing mechanism. The output score should be interpreted primarily as a model confidence or ranking score under the selected prompt context, rather than as a calibrated probability for all possible experimental settings. In practical screening, users may rank candidate sites by this score, whereas the decision threshold should be selected according to the expected application, available validation data, and the relative cost of false positives and false negatives. Routing weights and selected experts are provided to inspect how MOSAIC organizes internal cross-context support, but they should not be interpreted as direct biological similarity or mechanistic evidence. When the target species or methylation type is not represented in the benchmark, the query should be treated as an OOD use case. In such cases, the closest available prompt or a prompt sweep can be used as an exploratory strategy, but the resulting predictions should be interpreted cautiously and should not be treated as calibrated evidence for unsupported biological contexts.

The web server therefore serves not only as a deployment interface, but also as an interpretation layer for MOSAIC. By exposing expert routing alongside prediction outputs, it allows users to examine whether a prediction is driven primarily by dataset-specific specialization, species-related sharing, or methylation-type-related sharing. This capability is particularly valuable for multi-task biological prediction, where both practical usability and model transparency are important for downstream applications.

## Discussion

The present results show that, in heterogeneous methylation prediction, stronger benchmark-level performance does not necessarily translate into better generalization across tasks. Under severe cross-dataset imbalance, average gains can remain concentrated in favorable domains, while weak or underrepresented tasks benefit little. In this context, fairness is not merely a supplementary statistic, but an essential part of the evidence for whether cross-task learning is genuinely effective. The consistent improvements achieved by MOSAIC in worst MCC, head–tail disparity, and Jain’s fairness index indicate that it delivers better overall predictive performance, particularly by improving outcomes for tail tasks while maintaining strong benchmark-level performance.

These findings also help explain why traditional training paradigms are inadequate. Conventional multi-task learning shares information indiscriminately across all component datasets, including those with substantial biological heterogeneity and pronounced long-tailed imbalance. Because tasks differ in both resource level and biological context, such unrestricted sharing allows dominant datasets to exert a disproportionate influence on the shared representation space, while weaker tasks may receive transferred knowledge that is poorly aligned with their local structure. In contrast, MOSAIC handles knowledge transfer more selectively. By preserving a stable task-anchored route while recruiting auxiliary information only when beneficial, the model improves both predictive performance and cross-task balance without compromising low-resource tasks.

This interpretation is supported by evidence from ablation, routing, sequence-level, and perturbation analyses. The ablation results show that the gains of the full model do not arise from one single component alone, but from the coordinated effects of specialization, prompt-aware routing, sparse expert selection, and local refinement. The routing analysis further suggests that this organization is consistent with biologically plausible sequence and routing patterns: primary routing preserves dataset-specific structure, whereas auxiliary routing enables information sharing across related biological contexts. Sequence-level analyses indicate that stronger rank-specific expert dependence is associated with reproducible local sequence patterns, and perturbation experiments show that the auxiliary routes identified by the model are also functionally important. Together, these observations support an interpretable account in which MOSAIC preserves task identity while enabling sparse auxiliary transfer when related signal is available.

The external OOD results add a complementary perspective because they test whether the learned sharing strategy remains useful when the target context is absent from model training, rather than merely held out within an existing component dataset. The expanded evaluation indicates that MOSAIC can retain a degree of zero-shot transfer within the evaluated settings, but also shows that transfer is not a uniform property of all distribution shifts. Same-methylation-type transfer to unseen species was generally more reliable, suggesting that once the methylation chemistry is represented in the training benchmark, MOSAIC can reuse compatible sequence information across species through prompt-conditioned routing. By contrast, the 5mC setting was more difficult because the target methylation type itself was not represented during training. In that case, the useful signal came primarily from the 5hmC surrogate prompt, whereas unrelated methylation-type prompts were much less informative. This pattern is important because it suggests that MOSAIC does not simply respond to any available prompt under OOD shift; its transfer behavior is constrained by the biological and task context encoded in the prompt and routing structure. Thus, the OOD experiments strengthen the interpretation of MOSAIC as a selective-transfer framework, while indicating that the current evidence should be understood within the evaluated external settings.

Several limitations should be acknowledged. The present evaluation includes 20 component datasets and three methylation types, but it does not capture the full diversity of DNA modification systems. The external OOD analysis also suggests that missing methylation chemistry is a more severe source of shift than species mismatch within a known chemistry, indicating that broader type coverage will be important for future extension of the framework. In addition, although the interpretability analyses support a biologically plausible account of routing behavior, they do not establish a one-to-one correspondence between individual experts and specific molecular mechanisms. The fairness considered here is also task-level fairness under cross-dataset imbalance, rather than demographic or clinical fairness within a single dataset. Finally, the current model indicates that high-resource component datasets are the major donors in Top-2 expert recruitment in 6mA methylation, whereas phylogenetic proximity plays only a limited role. This pattern may reflect a limitation of the current representation, which does not explicitly incorporate phylogenetic information. Future work could therefore extend this framework to a broader taxonomic range, additional DNA modifications, more challenging OOD settings, richer representations that include phylogenetic structure, and more direct tests of causal biological interpretability.

Overall, MOSAIC reframes heterogeneous methylation prediction as a problem of capacity allocation under biological heterogeneity. By combining shared sequence modeling with prompt-aware sparse routing, the framework preserves task-specific specialization while enabling selective information sharing across related species and methylation contexts. The benchmark, external OOD, ablation, routing, and perturbation analyses together indicate that selective transfer provides a more effective and balanced strategy than indiscriminate sharing for this setting. The accompanying web server further makes MOSAIC available as a practical and inspectable tool for methylation prediction.

## Supplementary Material

gkag822_Supplemental_Files

## Data Availability

The datasets and trained model checkpoints used in this study are publicly available at https://github.com/Cpillar/MOSAIC and https://zenodo.org/records/19690524.
