# Supplementary material for: MOSAIC enables selective transfer and fairness-aware generalization in cross-species DNA methylation prediction
Source: Nucleic Acids Res. 2026 Aug 20;54(15):gkag822. doi: 10.1093/nar/gkag822 (PMC13490955; doi:10.1093/nar/gkag822)
Supplement: Supplementary file 1 [file gkag822_supplemental_files.zip › SIfigure.pdf]

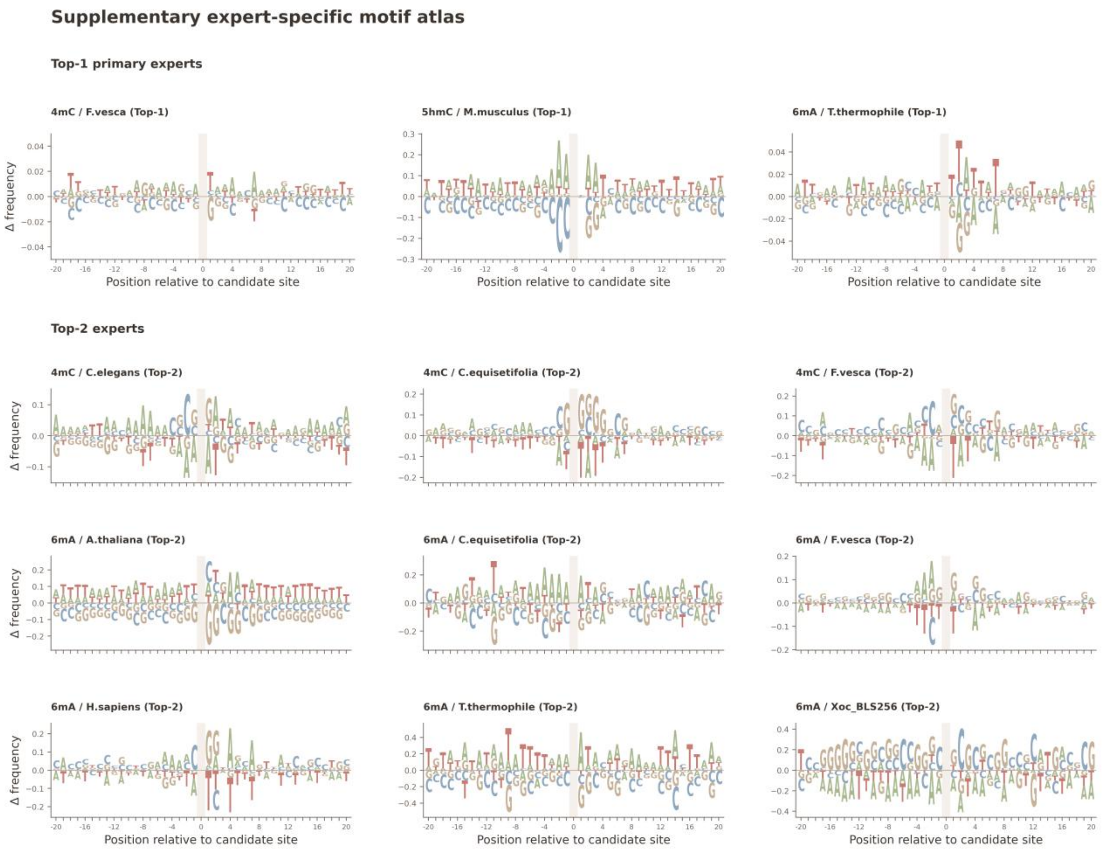

Caption： Figure S1. Supplementary expert-specific motif atlas for representative Top-1 and Top-2 experts in MOSAIC. Sequence logos show position-specific nucleotide preferences associated with representative experts identified from the routing analysis. The top row presents representative Top-1 primary experts, corresponding to the dominant task-anchored route for each benchmark, whereas the remaining panels present representative Top-2 auxiliary experts, reflecting secondary transfer patterns recruited under sparse expert routing. All logos were generated from 41-bp sequences centered on the candidate methylation site, with nucleotide enrichment and depletion shown relative to matched controls. Panel labels denote the methylation type, benchmark or species identity, and expert rank. Overall, the atlas supports the view that primary experts capture benchmark-dominant sequence context, whereas auxiliary experts encode more heterogeneous short-range sharing signals across related tasks.

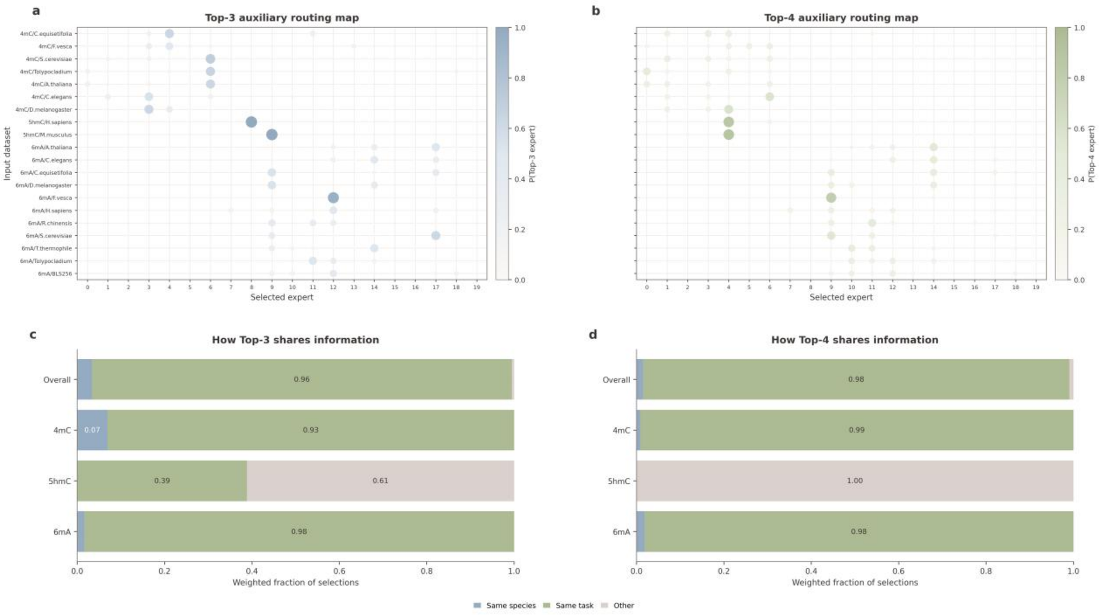

Caption：Figure S2. Supplementary analysis of higher-order auxiliary expert routing in MOSAIC. (a, b) Routing maps for the Top-3 and Top-4 auxiliary experts, respectively. Rows denote input benchmark datasets, columns denote selected experts, and bubble size/color intensity indicate the routing weight assigned to each expert. Compared with the Top-1 primary route and Top-2 auxiliary route discussed in the main text, Top-3 and Top-4 selections represent weaker but still structured secondary support channels. (c, d) Weighted composition of Top-3 and Top-4 expert sharing, grouped by whether the selected expert comes from the same species, the same benchmark task, or other tasks. The bars summarize the weighted fraction of auxiliary selections overall and within each methylation type. Top-3 and Top-4 routing is dominated by same-task reuse, with only limited same-species or cross-task sharing, suggesting that higher-order auxiliary routes provide residual task-stabilizing support rather than the main source of transferable information. This pattern is consistent with the routing hierarchy observed in MOSAIC, where the Top-1 route anchors task-specific specialization and lower-rank auxiliary routes contribute progressively weaker contextual sharing.

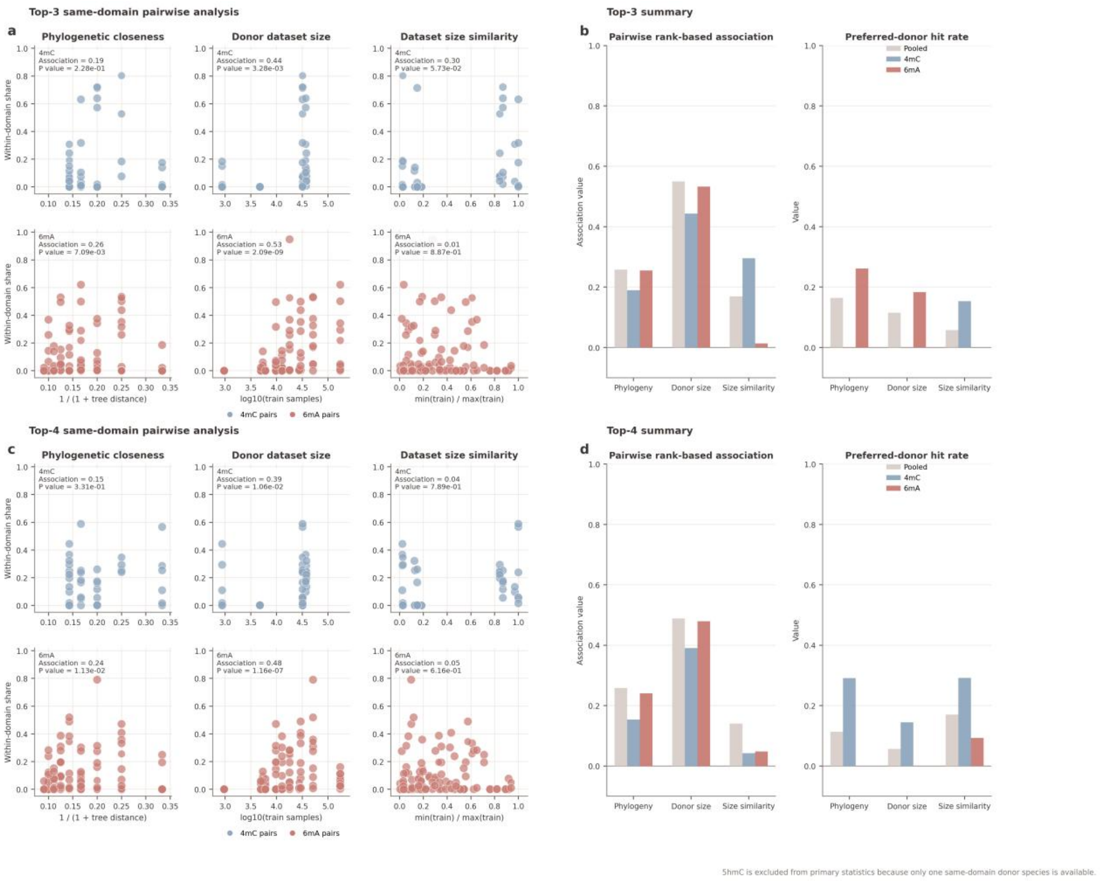

Caption: Figure S3. Supplementary pairwise analysis of same-domain donor preferences for higher-order auxiliary routing. (a, c) Pairwise analyses of Top-3 and Top-4 auxiliary expert sharing, respectively, within the same methylation domain. Each point represents a potential donor–recipient pair, and the y-axis shows the within-domain share assigned to the donor expert. Three explanatory factors are examined: phylogenetic closeness, donor dataset size, and dataset-size similarity. Analyses are shown separately for 4mC and 6mA pairs; 5hmC is excluded from the primary statistics because only one same-domain donor species is available. (b, d) Summary of rank-based associations and preferred-donor hit rates for Top-3 and Top-4 routing, respectively. Bar plots report the relative association between donor preference and each explanatory factor, together with the fraction of cases in which the preferred donor is consistent with the top-ranked candidate under each criterion. Across both Top-3 and Top-4 routes, donor dataset size shows the most consistent association with auxiliary donor preference, whereas phylogenetic closeness and dataset-size similarity contribute more weakly and less consistently. These results support the interpretation that higher-order auxiliary routes are dominated by residual same-domain support, with donor availability and dataset scale acting as stronger determinants than phylogenetic proximity alone.

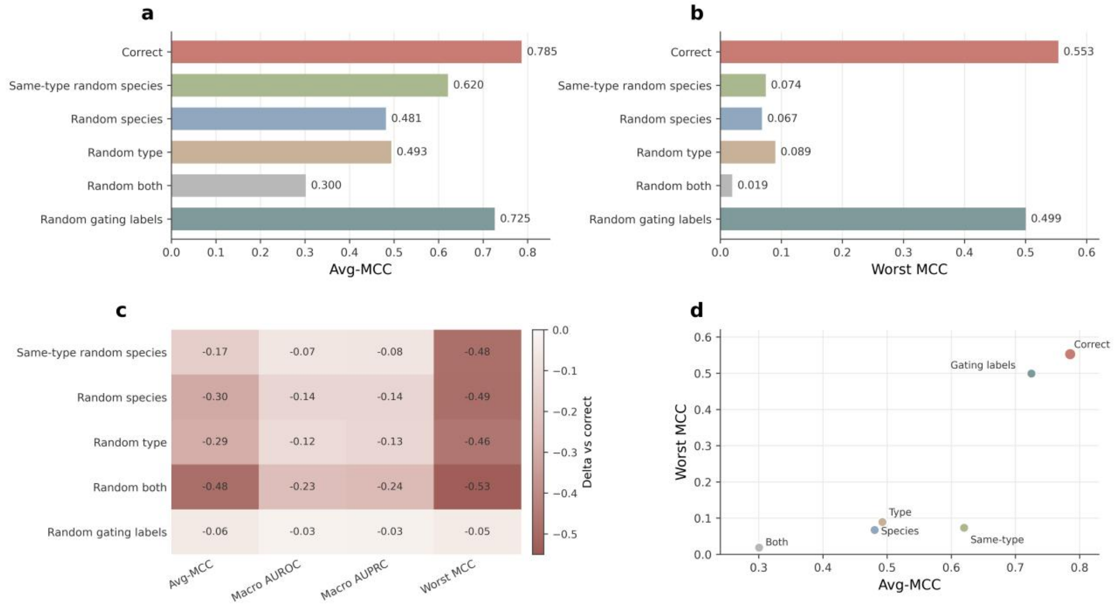

Caption: Figure S4. Prompt and routing-control analysis of MOSAIC. (a) Avg-MCC under the correct-prompt setting and under different prompt-control settings, including random species prompts, random methylation-type prompts, simultaneous randomization of both prompts, same-type random species prompts, and sample-wise random gating-label supervision. (b) Worst-dataset MCC under the same control settings. (c) Performance changes relative to the correct-prompt setting across Avg-MCC, macro AUROC, macro AUPRC, and worst-dataset MCC. (d) Joint robustness profile based on Avg-MCC and worst-dataset MCC. These controls perturb task-conditioning signals or routing supervision rather than removing model components. The performance decreases indicate that both prompt context and meaningful routing supervision contribute to the selective-transfer behavior of MOSAIC.

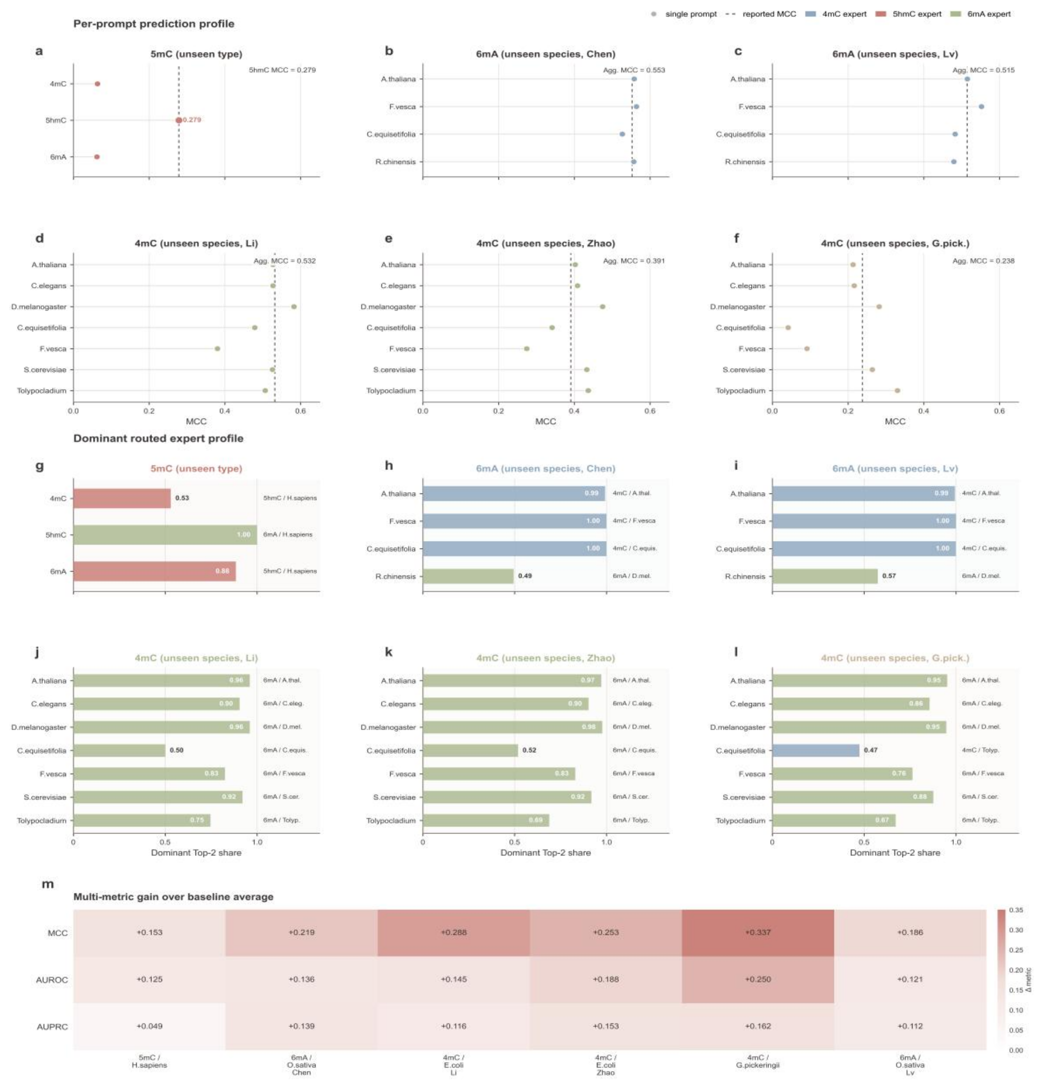

Caption: Figure S5. Prompt-level prediction and routing profiles under external OOD evaluation. (a-f) Per-prompt MCC profiles for the six external OOD datasets. For 5mC in H. sapiens, methylation-type prompts were swept across the available seen methylation types, and the 5hmC surrogate prompt was used as the reported MOSAIC score. For the 6mA and 4mC unseen-species datasets, species prompts were swept within the corresponding methylation-type family, and the dashed vertical line denotes the aggregate MCC used for the main OOD comparison. (g-l) Dominant Top-2 routed expert profiles for the same OOD datasets. Bars show the fraction of positive OOD samples assigned to the dominant Top-2 fallback expert under each swept prompt, and right-side labels indicate the corresponding routed expert. Expert colors denote the methylation-type family of the selected expert. Together, these profiles show that MOSAIC responds to OOD inputs through prompt-dependent fallback routes rather than indiscriminate expert averaging. (m) Dataset-level gains of the primary MOSAIC estimate over the mean of the evaluated public baselines for MCC, AUROC, and AUPRC. Each annotated cell shows MOSAIC minus the baseline average.
